# Supplementary material for: Analysis of retinal markers and incident amyotrophic lateral sclerosis: An optical coherence tomography-based cohort study
Source: PLoS Med. 2025 Jun 25;22(6):e1004545. doi: 10.1371/journal.pmed.1004545 (PMC12193630; doi:10.1371/journal.pmed.1004545)
Supplement: S1 Text — Fig A. The histogram of OCT parameters. Fig B. Correlation analysis between retinal markers, ocular diseases, and incident ALS risk. Table A. Code information for ocular diseases. Table B. Schoenfeld residuals test. Table C. Multicollinearity analysis. Table D. Normality test of retinal marker. Table E. Associations between retinal markers and incident ALS, additionally adjusted for smoking status. Table F. Associations between retinal markers and incident ALS, additionally additionally adjusted for maternal smoking around birth. Table G. Associations between retinal markers and incident ALS, excluding individuals with ocular disease at baseline and follow-up. Table H. Subgroup analysis. Table J. Logistic regression results between PRL and RPE with ocular disease disorders. Table K. Cox regression results between baseline ocular disease disorders and incident ALS risk. Methods. Obtaining of covariates. Analysis A. Relationships between PRL and RPE. Analysis B. OCT parameters and ALS subtype. Analysis C. PRL, RPE and time to ALS diagnosis. Analysis D. OCT parameters and brain MR images. Analysis E. MR analysis of OCT-related parameters and ALS. Analysis F. MR analysis of retinal vascular features and ALS. Analysis G. Retinal microvascular Changes and ALS. (PDF) [file pmed.1004545.s002.pdf]

Supplementary Files Catalogue

Fig A. The histogram of OCT parameters---2
Fig B. Correlation analysis between retinal markers, ocular diseases, and incident ALS risk. ------------3

Table A. Code information for ocular diseases. ---4
Table B. Schoenfeld residuals test. --5
Table C. Multicollinearity analysis. --5
Table D. Normality test of retinal markers--6
Table E. Associations between retinal markers and incident ALS, additionally adjusted for smoking status. --7
Table F. Associations between retinal markers and incident ALS, additionally additionally adjusted for maternal smoking around birth. --9
Table G. Associations between retinal markers and incident ALS, excluding individuals with ocular disease at baseline and follow-up. ---11
Table H. Subgroup analysis. --12
Table J. Logistic regression results between PRL and RPE with ocular disease disorders. ---------------13
Table K. Cox regression results between baseline ocular disease disorders and incident ALS risk. -----14

Methods. Obtaining of covariates.------------------------------------ ----------------------------------15

Analysis A Relationships between PRL and RPE--16
Analysis B OCT parameters and ALS subtype--17
Analysis C PRL, RPE and time to ALS diagnosis.--- ---19
Analysis D OCT parameters and brain MR images.---------------- --20
Analysis E MR analysis of OCT-related parameters and ALS --22
Analysis F MR analysis of retinal vascular features and ALS--23
Analysis G Retinal microvascular Changes and ALS ---24

Fig A. The histogram of OCT parameters.

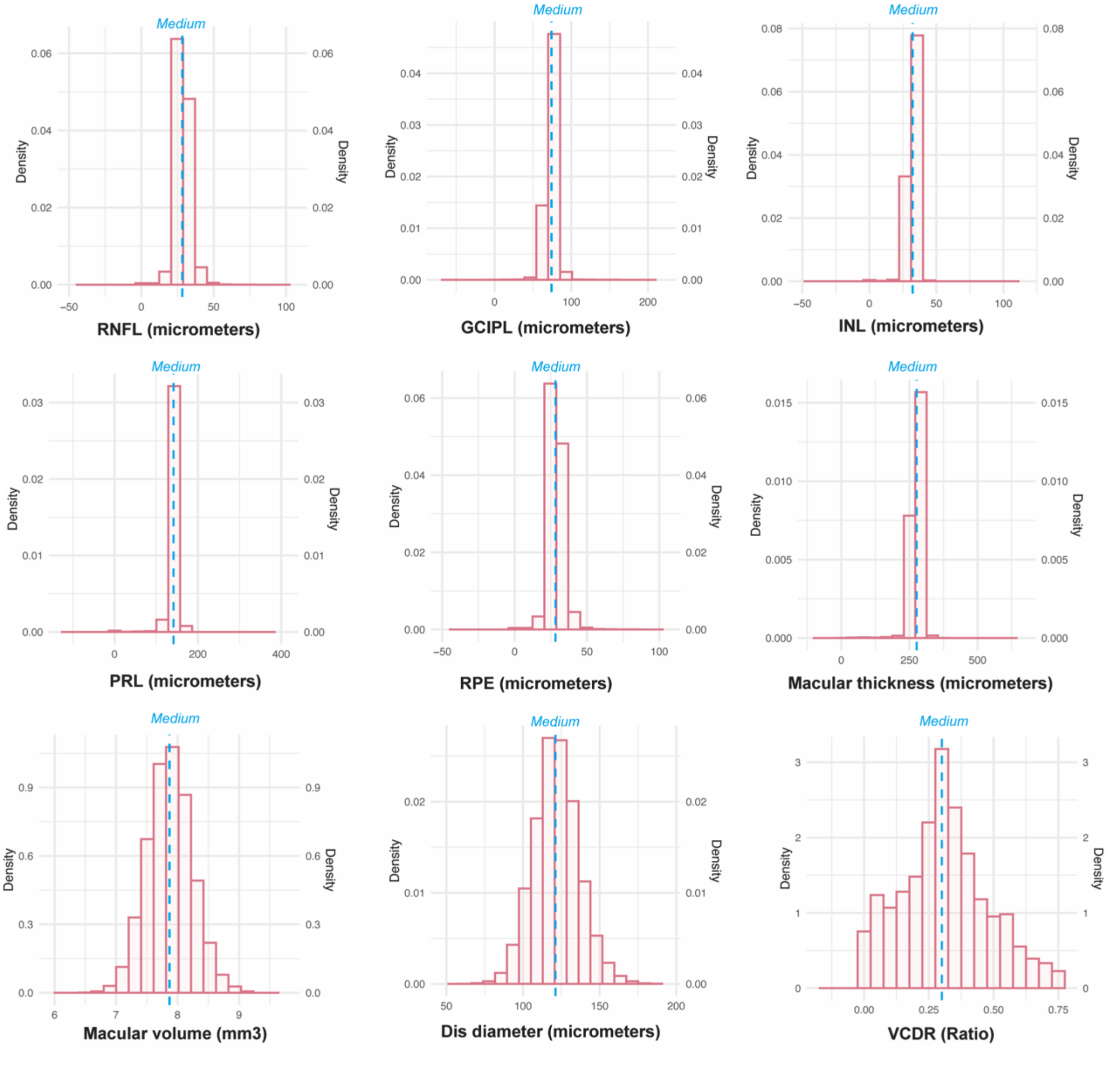

Abbreviation: OCT, Optical coherence tomography; RNFL, Retinal nerve fiber layer; GCIPLT, Ganglion cell-inner plexiform layer thickness; INL, Inner nuclear layer; PRL, Photoreceptor layer; RPE, Retinal pigment epithelium; VCDR, Vertical cup-to-disc ratio.

Fig B. Correlation analysis between retinal markers, ocular diseases, and incident ALS risk.

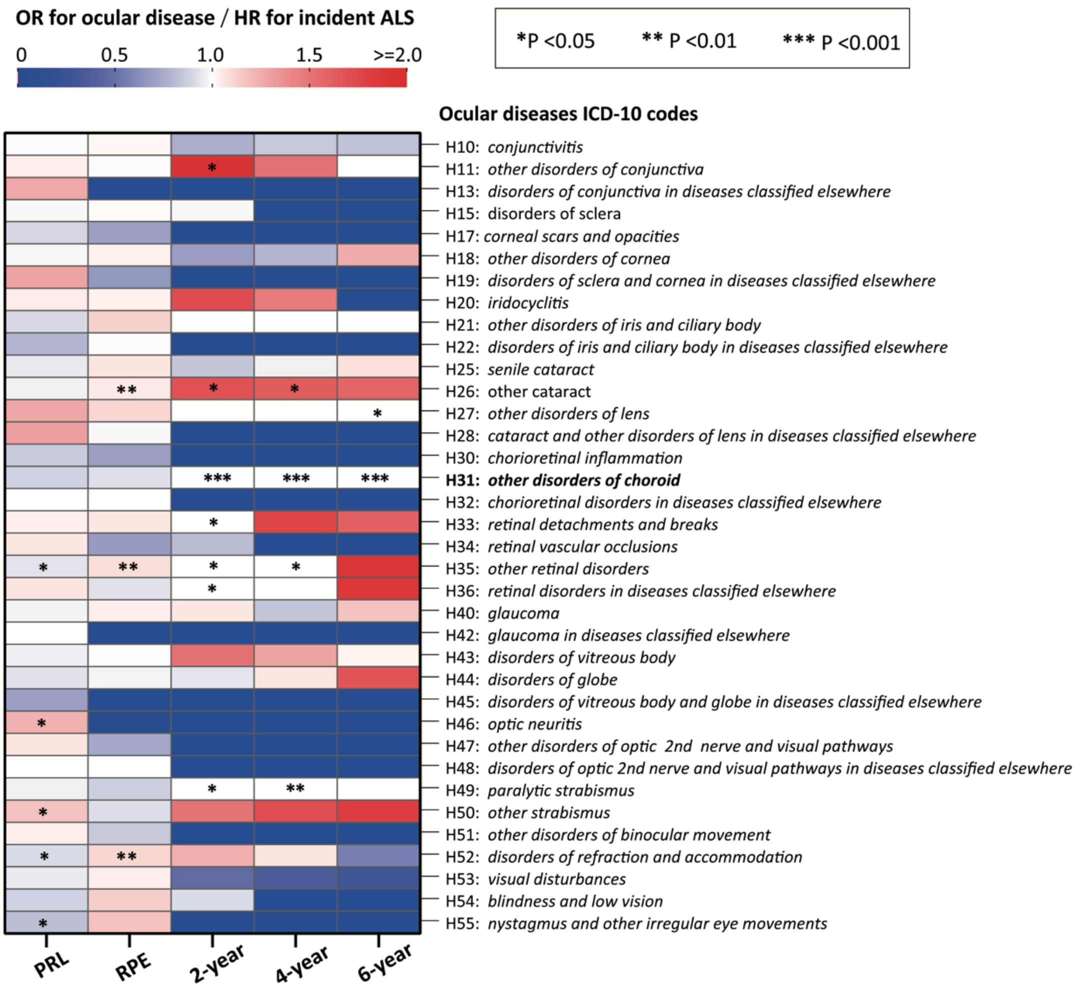

Adjusted for age, sex, ethnicity, TDI, hypertension, diabetes, intraocular pressure, and spherical equivalent

Abbreviation: TDI, Townsend Deprivation Index; BMI, Body mass index; PRL, Photoreceptor layer; RPE, Retinal pigment epithelium.

Table A. Code information for ocular diseases.

ICD-10 codes	Diseases
H10	conjunctivitis
H11	other disorders of conjunctiva
H13	disorders of conjunctiva in diseases classified elsewhere
H15	disorders of sclera
H17	corneal scars and opacities
H18	other disorders of cornea
H19	disorders of sclera and cornea in diseases classified elsewhere
H20	iridocyclitis
H21	other disorders of iris and ciliary body
H22	disorders of iris and ciliary body in diseases classified elsewhere
H25	senile cataract
H26	other cataract
H27	other disorders of lens
H28	cataract and other disorders of lens in diseases classified elsewhere
H30	Chori retinal inflammation
H31	other disorders of choroid
H32	Chori retinal disorders in diseases classified elsewhere
H33	retinal detachments and breaks
H34	retinal vascular occlusions
H35	other retinal disorders
H36	retinal disorders in diseases classified elsewhere
H40	glaucoma
H42	glaucoma in diseases classified elsewhere
H43	disorders of vitreous body
H44	disorders of globe
H45	disorders of vitreous body and globe in diseases classified elsewhere
H46	optic neuritis
H47	other disorders of optic 2nd nerve and visual pathways
H48	disorders of optic 2nd nerve and visual pathways in diseases classified elsewhere
H49	paralytic strabismus
H50	other strabismus
H51	other disorders of binocular movement
H52	disorders of refraction and accommodation
H53	visual disturbances
H54	blindness and low vision
H55	nystagmus and other irregular eye movements

Table B. Schoenfeld residuals test.

Adjusted model: adjusted by age, sex, ethnicity, TDI, hypertension, diabetes, intraocular pressure, and spherical equivalent;

Sensitivity analysis 1: Excluding participants with a follow up less than 4 years;

Sensitivity analysis 2: Excluding participants with a follow up less than 6 years

	**Schoenfeld residuals test p value**			
	Unadjusted model	Adjusted model	Sensitivity 1	Sensitivity 2
RNFL	0.873	0.886	0.289	0.792
GCIPLT	0.671	0.620	0.640	**NS**
INL	0.441	0.432	0.561	0.187
PRL	0.547	0.565	0.894	0.689
RPE	0.522	0.547	0.899	0.666
Macular thickness	0.918	0.925	0.601	0.776
Macular volume	0.094	0.918	0.085	**0.020**
Disc diameter	0.983	0.987	0.681	0.303
VCDR	0.319	0.315	0.317	0.064

Abbreviation: RNFL, Retinal nerve fiber layer; GCIPLT, Ganglion cell-inner plexiform layer thickness; INL, Inner nuclear layer; PRL, Photoreceptor layer; RPE, Retinal pigment epithelium; VCDR, Vertical cup-to-disc ratio.

Table C. Multicollinearity analysis.

	Variable								
VIF	RNFL	GCIPL	INL	PRL	RPE	Macular thickness	Macular volume	Disc diameter	VCDR
Variable	1.028	1.025	1.017	1.004	1.006	1.007	1.032	1.068	1.012
Age	1.018	1.019	1.023	1.019	1.019	1.019	1.017	1.021	1.021
Sex	1.021	1.016	1.017	1.018	1.016	1.018	1.011	1.019	1.019
TDI	1.052	1.052	1.053	1.054	1.054	1.053	1.028	1.058	1.058
Ethnic	1.060	1.060	1.061	1.061	1.061	1.060	1.033	1.068	1.068
Diabetes	1.071	1.071	1.071	1.070	1.071	1.070	1.000	1.067	1.066
Hypertension	1.071	1.074	1.071	1.070	1.070	1.071	1.017	1.068	1.067
Spherical equivalent	1.023	1.021	1.013	1.005	1.008	1.007	1.026	1.069	1.012
Intraocular pressure	1.005	1.009	1.009	1.007	1.007	1.006	1.006	1.008	1.010

Abbreviation: TDI, Townsend Deprivation Index; BMI, Body mass index; RNFL, Retinal nerve fiber layer; GCIPLT, Ganglion cell-inner plexiform layer thickness; INL, Inner nuclear layer; PRL, Photoreceptor layer; RPE, Retinal pigment epithelium; VCDR, Vertical cup-to-disc ratio.

Table D. Normality test of retinal markers.

Variable	Kolmogorov-Smirnov p value	Anderson-Darling p value
PRL	**<0.001**	**<0.001**
GCIPL	**<0.001**	**<0.001**
INL	**<0.001**	**<0.001**
RNFL	**<0.001**	**<0.001**
Disc diameter	**<0.001**	**<0.001**
RPE	**<0.001**	**<0.001**
Macular thickness	**<0.001**	**<0.001**
Macular volume	0.071	**<0.001**
VCDR	**<0.001**	**<0.001**

Abbreviation: RNFL, Retinal nerve fiber layer; GCIPLT, Ganglion cell-inner plexiform layer thickness; INL, Inner nuclear layer; PRL, Photoreceptor layer; RPE, Retinal pigment epithelium; VCDR, Vertical cup-to-disc ratio.

Table E. Associations between retinal markers and incident ALS, additionally adjusted for smoking status.

Incident ALS	Models	Per SD increase HR (95%CI)	p
RNFL (micrometers)	Unadjusted	1.157 (0.875,1.529)	0.3071
	Adjusted	1.238 (0.977,1.568)	0.0773
	Sensitivity analysis 1	1.126 (0.800,1.585)	0.4966
	Sensitivity analysis 2	1.104 (0.902,1.352)	0.3359
GCIPL (micrometers)	Unadjusted	1.103 (0.888,1.370)	0.3743
	Adjusted	1.103 (0.868,1.403)	0.4220
	Sensitivity analysis 1	**1.160 (1.039,1.295)**	**0.0082**
	Sensitivity analysis 2	1.048 (0.794,1.383)	0.7402
INL (micrometers)	Unadjusted	1.018 (0.737,1.405)	0.9142
	Adjusted	**0.812 (0.711,0.928)**	**0.0023** *
	Sensitivity analysis 1	**0.845 (0.719,0.993)**	**0.0404**
	Sensitivity analysis 2	**0.821 (0.697,0.966)**	**0.0178**
PRL (micrometers)	Unadjusted	**1.199 (1.069,1.345)**	**0.0019***
	Adjusted	**1.158 (1.006,1.334)**	**0.0414**
	Sensitivity analysis 1	**1.190 (1.034,1.369)**	**0.0152**
	Sensitivity analysis 2	0.888 (0.728,1.083)	0.2397
RPE (micrometers)	Unadjusted	0.927 (0.731,1.176)	0.5340
	Adjusted	0.876 (0.699,1.097)	0.2488
	Sensitivity analysis 1	0.826 (0.582,1.171)	0.2823
	Sensitivity analysis 2	0.825 (0.578,1.176)	0.2867
Macular thickness (micrometers)	Unadjusted	0.797 (0.532,1.194)	0.2716
	Adjusted	0.876 (0.664,1.154)	0.3463
	Sensitivity analysis 1	0.855 (0.641,1.141)	0.2865
	Sensitivity analysis 2	0.986 (0.719,1.352)	0.9286
Macular volume (mm3)	Unadjusted	0.907 (0.694,1.185)	0.4723
	Adjusted	0.895 (0.678,1.183)	0.4364
	Sensitivity analysis 1	0.924 (0.681,1.253)	0.6100
	Sensitivity analysis 2	1.157 (0.875,1.529)	0.3071
Disc diameter (micrometers)	Unadjusted	1.238 (0.977,1.568)	0.0773
	Adjusted	1.126 (0.800,1.585)	0.4966
	Sensitivity analysis 1	1.104 (0.902,1.352)	0.3359
	Sensitivity analysis 2	1.103 (0.888,1.370)	0.3743
VCDR (ratio)	Unadjusted	1.103 (0.868,1.403)	0.4220
	Adjusted	**1.160 (1.039,1.295)**	**0.0082**
	Sensitivity analysis 1	1.048 (0.794,1.383)	0.7402
	Sensitivity analysis 2	1.018 (0.737,1.405)	0.9142

Adjusted: Adjusted for age, sex, ethnicity, TDI, hypertension, diabetes, intraocular pressure, spherical equivalent and smoking status; Sensitivity analysis 1: Excluding participants with a follow up less than 4 years; Sensitivity analysis 2: Excluding participants with a follow up less than 6 years. *significant after Bonferroni correction.

Abbreviation: ALS, Amyotrophic lateral sclerosis; TDI, Townsend Deprivation Index; BMI, Body mass

index; IOP, Intraocular pressure; RNFL, Retinal nerve fiber layer; GCIPLT, Ganglion cell-inner plexiform layer thickness; INL, Inner nuclear layer; PRL, Photoreceptor layer; RPE, Retinal pigment epithelium; SD, Standard deviation; VCDR, Vertical cup-to-disc ratio.

Table F. Associations between retinal markers and incident ALS, additionally additionally adjusted for maternal smoking around birth.

Incident ALS	Models	Per SD increase HR (95%CI)	p
RNFL (micrometers)	Unadjusted	1.148 (0.867,1.521)	0.3344
	Adjusted	1.226 (0.964,1.560)	0.0973
	Sensitivity analysis 1	1.113 (0.787,1.574)	0.5462
	Sensitivity analysis 2	1.101 (0.897,1.351)	0.3578
GCIPL (micrometers)	Unadjusted	1.097 (0.878,1.371)	0.4143
	Adjusted	1.097 (0.857,1.405)	0.4622
	Sensitivity analysis 1	**1.157 (1.037,1.290)**	**0.0092**
	Sensitivity analysis 2	1.047 (0.795,1.378)	0.7443
INL (micrometers)	Unadjusted	1.017 (0.738,1.401)	0.9188
	Adjusted	**0.813 (0.712,0.929)**	**0.0024** *
	Sensitivity analysis 1	**0.846 (0.720,0.995)**	**0.0432**
	Sensitivity analysis 2	**0.822 (0.698,0.968)**	**0.0189**
PRL (micrometers)	Unadjusted	**1.198 (1.068,1.343)**	**0.0020** *
	Adjusted	**1.157 (1.005,1.333)**	**0.0427**
	Sensitivity analysis 1	**1.189 (1.034,1.368)**	**0.0155**
	Sensitivity analysis 2	0.887 (0.728,1.080)	0.2331
RPE (micrometers)	Unadjusted	0.925 (0.731,1.171)	0.5168
	Adjusted	0.874 (0.699,1.094)	0.2391
	Sensitivity analysis 1	0.826 (0.582,1.174)	0.2865
	Sensitivity analysis 2	0.824 (0.577,1.176)	0.2861
Macular thickness (micrometers)	Unadjusted	0.796 (0.531,1.194)	0.2706
	Adjusted	0.878 (0.666,1.157)	0.3552
	Sensitivity analysis 1	0.857 (0.642,1.143)	0.2936
	Sensitivity analysis 2	0.988 (0.720,1.354)	0.9385
Macular volume (mm3)	Unadjusted	0.907 (0.694,1.185)	0.4754
	Adjusted	0.896 (0.679,1.184)	0.4416
	Sensitivity analysis 1	0.924 (0.681,1.255)	0.6130
	Sensitivity analysis 2	1.148 (0.867,1.521)	0.3344
Disc diameter (micrometers)	Unadjusted	1.226 (0.964,1.560)	0.0973
	Adjusted	1.113 (0.787,1.574)	0.5462
	Sensitivity analysis 1	1.101 (0.897,1.351)	0.3578
	Sensitivity analysis 2	1.097 (0.878,1.371)	0.4143
VCDR (ratio)	Unadjusted	1.097 (0.857,1.405)	0.4622
	Adjusted	**1.157 (1.037,1.290)**	**0.0092**
	Sensitivity analysis 1	1.047 (0.795,1.378)	0.7443
	Sensitivity analysis 2	1.017 (0.738,1.401)	0.9188

Adjusted: Adjusted for age, sex, ethnicity, TDI, hypertension, diabetes, intraocular pressure, spherical equivalent and maternal smoking around birth; Sensitivity analysis 1: Excluding participants with a follow up less than 4 years; Sensitivity analysis 2: Excluding participants with a follow up less than 6 years.

*significant after Bonferroni correction.

Abbreviation: ALS, Amyotrophic lateral sclerosis; TDI, Townsend Deprivation Index; BMI, Body mass index; IOP, Intraocular pressure; RNFL, Retinal nerve fiber layer; GCIPLT, Ganglion cell-inner plexiform layer thickness; INL, Inner nuclear layer; PRL, Photoreceptor layer; RPE, Retinal pigment epithelium; SD, Standard deviation; VCDR, Vertical cup-to-disc ratio.

Table G. Associations between retinal markers and incident ALS, excluding individuals with ocular disease at baseline and follow-up.

Incident ALS	Models	Per SD increase HR (95%CI)	p
RNFL	Unadjusted	1.09 (0.73,1.62)	0.683
(micrometers)	Adjusted	1.13 (0.77,1.66)	0.535
	Sensitivity analysis 1	1.26 (0.95,1.65)	0.104
	Sensitivity analysis 2	1.09 (0.73,1.62)	0.683
GCIPL	Unadjusted	**1.18 (1.01,1.39)**	**0.042**
(micrometers)	Adjusted	1.18 (0.99,1.41)	0.063
	Sensitivity analysis 1	1.18 (0.97,1.43)	0.100
	Sensitivity analysis 2	1.19 (0.97,1.46)	0.097
INL	Unadjusted	**1.17 (1.05,1.31)**	**0.005** *
(micrometers)	Adjusted	**1.17 (1.04,1.31)**	**0.008**
	Sensitivity analysis 1	0.96 (0.67,1.40)	0.849
	Sensitivity analysis 2	0.95 (0.63,1.41)	0.785
PRL	Unadjusted	**0.77 (0.67,0.89)**	**0.000** *
(micrometers)	Adjusted	**0.76 (0.66,0.88)**	**0.000** *
	Sensitivity analysis 1	**0.79 (0.67,0.95)**	**0.009**
	Sensitivity analysis 2	**0.76 (0.64,0.90)**	**0.002** *
RPE	Unadjusted	**1.26 (1.12,1.41)**	**0.000** *
(micrometers)	Adjusted	**1.27 (1.13,1.43)**	**0.000** *
	Sensitivity analysis 1	**1.23 (1.07,1.42)**	**0.004** *
	Sensitivity analysis 2	**1.27 (1.10,1.46)**	**0.001** *
Macular thickness	Unadjusted	0.85 (0.68,1.07)	0.170
(micrometers)	Adjusted	0.84 (0.67,1.04)	0.112
	Sensitivity analysis 1	0.88 (0.67,1.15)	0.340
	Sensitivity analysis 2	0.82 (0.64,1.05)	0.122
Macular volume	Unadjusted	0.86 (0.55,1.32)	0.484
(mm3)	Adjusted	0.89 (0.57,1.39)	0.603
	Sensitivity analysis 1	0.89 (0.57,1.39)	0.603
	Sensitivity analysis 2	0.89 (0.55,1.44)	0.627
Disc diameter	Unadjusted	0.87 (0.62,1.21)	0.396
(micrometers)	Adjusted	0.84 (0.60,1.19)	0.324
	Sensitivity analysis 1	0.80 (0.56,1.15)	0.230
	Sensitivity analysis 2	1.03 (0.70,1.52)	0.893
VCDR	Unadjusted	0.89 (0.63,1.24)	0.477
(ratio)	Adjusted	0.88 (0.63,1.24)	0.469
	Sensitivity analysis 1	0.84 (0.58,1.20)	0.329
	Sensitivity analysis 2	0.84 (0.57,1.24)	0.383

Adjusted for age, sex, ethnicity, TDI, hypertension, diabetes, intraocular pressure, and spherical equivalent; Sensitivity analysis 1: Excluding participants with a follow up less than 4 years; Sensitivity analysis 2: Excluding participants with a follow up less than 6 years.

*significant after Bonferroni correction.

Abbreviation: ALS, Amyotrophic lateral sclerosis; TDI, Townsend Deprivation Index; BMI, Body mass

index; IOP, Intraocular pressure; RNFL, Retinal nerve fiber layer; GCIPLT, Ganglion cell-inner plexiform layer thickness; INL, Inner nuclear layer; PRL, Photoreceptor layer; RPE, Retinal pigment epithelium; SD, Standard deviation; VCDR, Vertical cup-to-disc ratio.

Table H. Subgroup analysis.

	**PRL**				**RPE**			
	Sample size	HR (95%CI)	p	p for interaction	Sample size	HR (95%CI)	p	p for interaction
**Age**				0.329				0.354
<60 year	29265	0.87 (0.70,1.08)	0.209		29265	1.13 (0.93,1.37)	0.218	
>=60 year	22434	0.77 (0.65,0.91)	0.002		22434	1.26 (1.09,1.45)	0.002	
**Sex**				0.494				0.669
Female	28456	0.78 (0.67,0.92)	0.003		28456	1.23 (1.07,1.41)	0.004	
Male	23243	0.86 (0.67,1.09)	0.218		23243	1.17 (0.95,1.43)	0.133	
**Ethnic**				**0.027**				0.377
Non,white	2809	7.24 (0.84,62.53)	0.072		2809	0.06 (0,2630.63)	0.609	
White	48890	0.80 (0.70,0.91)	0.001		48890	1.21 (1.08,1.36)	0.001	
**TDI**								0.381
<,2.120	26092	0.85 (0.68,1.06)	0.141	0.500	26092	1.14 (0.92,1.40)	0.230	
>=,2.120	25607	0.78 (0.66,0.92)	0.004		25607	1.24 (1.08,1.43)	0.002	
**Hypertension**				0.276				0.139
No	38292	0.79 (0.69,0.90)	0.000		38292	1.23 (1.10,1.38)	0.000	
Yes	13407	1.08 (0.68,1.72)	0.744		13407	0.36 (0,37.56)	0.669	
**Diabetes**				0.627				0.246
No	49065	0.80 (0.70,0.92)	0.002		49065	1.21 1.08,1.36)	0.001	
Yes	2634	0.92 (0.46,1.85)	0.822		2634	0.02 (0,292.18)	0.434	
**BMI**				0.611				0.755
<25	17034	0.77 (0.62,0.96)	0.020		17034	1.23 (1.02,1.49)	0.033	
>=25	34408	0.83 (0.70,0.98)	0.032		34408	1.19 (1.03,1.37)	0.018	
**Smoke**				**0.018**				**0.024**
no	28261	1.12 0.79,1.60)	0.512		28261	0.74 (0.22,2.50)	0.626	
yes	23127	0.74 (0.64,0.84)	0.000		23127	1.29 (1.15,1.45)	0.000	

Adjusted for age, sex, ethnicity, TDI, hypertension, diabetes, intraocular pressure, and spherical equivalent.

Abbreviation: TDI, Townsend Deprivation Index; BMI, Body mass index; PRL, Photoreceptor layer; RPE, Retinal pigment epithelium.

Table J. Logistic regression results between PRL and RPE with ocular disease disorders.

**Disease code**	**PRL**				**RPE**			
	OR	lower 95%CI	Upper 95%CI	P value	OR	lower 95%CI	Upper 95%CI	P value
H10	0.990	0.948	1.034	0.660	1.013	0.972	1.056	0.543
H11	1.038	0.949	1.136	0.415	0.989	0.905	1.080	0.801
H13	1.269	0.681	2.364	0.452	0.005	0.000	3890.274	0.449
H15	0.983	0.825	1.172	0.851	1.008	0.846	1.201	0.930
H17	0.898	0.576	1.399	0.634	0.697	0.083	5.857	0.740
H18	0.979	0.831	1.152	0.794	1.031	0.887	1.199	0.689
H19	1.289	0.907	1.831	0.157	0.663	0.007	60.549	0.858
H20	1.047	0.907	1.208	0.533	1.030	0.917	1.157	0.618
H21	0.898	0.684	1.180	0.441	1.124	0.903	1.399	0.297
H22	0.787	0.282	2.192	0.646	0.990	0.112	8.764	0.993
H25	0.944	0.849	1.049	0.285	1.066	0.972	1.170	0.176
H26	0.967	0.931	1.004	0.084	1.048	1.014	1.084	**0.006**
H27	1.274	0.971	1.672	0.080	1.114	0.799	1.553	0.526
H28	1.305	0.923	1.844	0.132	0.982	0.384	2.509	0.970
H30	0.856	0.614	1.191	0.356	0.693	0.103	4.674	0.706
H31	0.884	0.705	1.107	0.282	0.919	0.576	1.464	0.721
H32	1.000	0.000	Inf	1.000	1.000	0.000	Inf	1.000
H33	1.036	0.934	1.150	0.506	1.057	0.979	1.141	0.157
H34	1.055	0.865	1.287	0.595	0.676	0.341	1.341	0.263
H35	0.928	0.869	0.990	**0.024**	1.084	1.025	1.147	**0.005**
H36	1.061	0.945	1.191	0.316	0.922	0.796	1.067	0.277
H40	0.972	0.920	1.026	0.300	1.039	0.989	1.092	0.128
H42	3.036	0.886	10.403	0.077	0.010	0.000	196656205.500	0.704
H43	0.960	0.892	1.033	0.271	1.001	0.924	1.085	0.974
H44	0.925	0.798	1.073	0.304	0.976	0.796	1.196	0.814
H45	0.703	0.335	1.477	0.352	0.000	0.000	291.012	0.153
H46	1.231	1.049	1.445	**0.011**	0.037	0.001	1.019	0.051
H47	1.061	0.817	1.378	0.658	0.733	0.349	1.539	0.411
H48	1.000	0.000	Inf	1.000	1.000	0.000	Inf	1.000
H49	0.963	0.720	1.287	0.797	0.866	0.484	1.548	0.627
H50	1.174	1.029	1.341	**0.018**	0.917	0.737	1.142	0.440
H51	1.039	0.674	1.601	0.862	0.851	0.397	1.821	0.677
H52	0.907	0.828	0.993	**0.035**	1.106	1.025	1.194	**0.010**
H53	0.947	0.885	1.014	0.120	1.038	0.972	1.110	0.268
H54	0.880	0.754	1.028	0.108	1.135	0.999	1.290	0.052
H55	0.814	0.665	0.997	**0.047**	1.180	0.985	1.415	0.073

Abbreviation: PRL, Photoreceptor layer; RPE, Retinal pigment epithelium.

Table K. Cox regression results between baseline ocular disease disorders and incident ALS risk.

Disease code	2-year landmark		4-year landmark		6-year landmark	
	HR	P value	HR	P value	HR	P value
H10	0.750	0.256	0.850	0.523	0.827	0.555
H11	1.917	**0.041**	1.515	0.275	2.079	0.076
H13	0.000	0.988	0.000	0.989	0.000	0.991
H15	0.983	0.986	0.000	0.985	0.000	0.988
H17	0.000	0.983	0.000	0.984	0.000	0.987
H18	0.690	0.711	0.783	0.807	1.258	0.819
H19	0.000	0.987	0.000	0.988	0.000	0.990
H20	1.725	0.277	1.466	0.509	0.000	0.988
H21	2.575	0.345	2.935	0.282	4.731	0.121
H22	0.000	0.990	0.000	0.990	0.000	0.992
H25	0.841	0.764	0.964	0.949	1.072	0.922
H26	1.694	**0.010**	1.637	**0.027**	1.592	0.115
H27	4.287	0.146	4.871	0.114	7.897	**0.039**
H28	0.000	0.990	0.000	0.990	0.000	0.992
H30	0.000	0.984	0.000	0.985	0.000	0.988
H31	11.898	**0.000**	13.572	**0.000**	16.409	**0.000**
H32	0.000	0.990	0.000	0.990	0.000	0.992
H33	2.221	**0.012**	1.760	0.138	1.614	0.341
H34	0.808	0.831	0.000	0.984	0.000	0.988
H35	2.036	**0.015**	2.128	**0.013**	1.891	0.122
H36	2.431	**0.048**	2.235	0.109	1.872	0.377
H40	1.057	0.863	0.841	0.650	1.176	0.694
H42	0.000	0.991	0.000	0.992	0.001	0.991
H43	1.519	0.240	1.286	0.540	1.027	0.964
H44	0.933	0.945	1.056	0.956	1.680	0.604
H45	0.000	0.990	0.000	0.990	0.000	0.993
H46	0.000	0.984	0.000	0.985	0.000	0.988
H47	0.000	0.981	0.000	0.982	0.000	0.986
H48	0.000	0.990	0.000	0.990	0.000	0.992
H49	6.011	**0.011**	6.909	**0.006**	5.658	0.084
H50	1.512	0.475	1.716	0.351	1.826	0.396
H51	0.000	0.985	0.000	0.985	0.000	0.989
H52	1.251	0.656	1.063	0.916	0.568	0.573
H53	0.464	0.184	0.350	0.138	0.282	0.206
H54	0.909	0.924	0.000	0.985	0.000	0.988
H55	0.000	0.982	0.000	0.983	0.000	0.987

Methods: Obtaining of covariates.

The following describes the specific collection information for the covariates included in the study. Observations with any missing values of the covariates were excluded from this study.

Baseline characteristics: Sociodemographic factors, including age, sex, ethnicity, BMI, and TDI, were obtained during the initial assessment. Body-mass index (BMI) was calculated as weight/height. Townsend Deprivation Index was derived from the national census, data on unemployment, ownership of vehicles, household overcrowding, and occupation. Smoking status were self-reported. Medication history, encompassing conditions such as hypertension (ICD-10 codes: "I10," "I15," "I11," "I19," "I16"), diabetes ("E10," "E11," "O24," "E13") was extracted from the ICD-10 codes.

Refractometry: The spherical equivalent is calculated by adding the spherical power to half of the cylindrical power. Lens refractometry measures the participant's current eye prescription, providing refractometry outputs (Sphere, Cylinder, Axis, Pupil diameter) and keratometry outputs (corneal refraction and astigmatism). The instrument used for this measurement is the Tomey RC-5000 (Tomey, USA).

Intraocular pressure: Intraocular pressure can be screened using an optical response analyzer, which measures the difference in air pressures at which the cornea flattens inward and outward. This is done using a gentle puff of air, ensuring no contact with the eye. The instrument used at the UK Biobank Assessment Centre for this measurement is the Reichert Ocular Response Analyzer (ORA).

Analysis A: Relationships between PRL and RPE.

Our study identified two meaningful indicators, and further exploration of their relationship, as well as their potential synergistic or independent effects on the outcomes, would be highly valuable. Thus, we have additionally conducted the corresponding statistical analyses:

(1) PRL and RPE remained significant in the individual analyses. However, when both factors were included in the multivariable regression model (adjusted for age, sex, ethnicity, Townsend Deprivation Index, hypertension, diabetes, intraocular pressure, and spherical equivalent), their significance diminished:

PRL: HR (95% CI) = 0.88 (0.55–1.41), P = 0.597.

RPE: HR (95% CI) = 1.08 (0.71–1.63), P = 0.724.

Further analysis revealed a strong negative correlation between PRL and RPE (r = -0.799, 95% CI: -0.8012 to -0.796, P = 2.2×10^{-16}). Additionally, as shown in the table below, high multicollinearity was observed in the multivariable model (Variance inflation factor, VIF > 10), suggesting that their effects may be difficult to separate statistically. Interaction analysis indicated no significant interaction between PRL and RPE (P for interaction = 0.235).

Multicollinearity analysis in multivariable regression model.

Index	VIF	Tolerance (1/VIF)
RPE	**13.086**	0.076
PRL	**13.069**	0.077
Age	1.019	0.982
Sex	1.034	0.967
TDI	1.054	0.949
Ethnic	1.061	0.943
Diabetes	1.076	0.930
Hypertension	1.073	0.932
Spherical equivalent	1.036	0.965
Intraocular pressure	1.008	0.992

Abbreviation: Variance inflation factor, VIF; PRL, Photoreceptor layer; RPE, Retinal pigment epithelium; TDI, Townsend Deprivation Index.

Thus, due to the strong correlation between PRL and RPE, they are likely measuring the same variation in risk. When comparing these two indices and considering VIF and AIC/BIC model fit indicators, PRL may be the preferred variable to retain. Future studies should further evaluate the validity of both factors based on biological significance and explore their potential independence in influencing risk.

Evaluation of PRL and RPE in multivariable regression model.

Index	PRL	RPE
VIF	13.069	13.086
AIC	1475.646	1475.812
BIC	1500.060	1500.227

Abbreviation: Variance inflation factor, VIF AIC, Akaike Information Criterion; BIC, Bayesian Information Criterion; PRL, Photoreceptor layer; RPE, Retinal pigment epithelium.

Analysis B: OCT parameters and ALS subtype.

There is no detailed clinical information on ALS in UK biobank. Due to the low incidence rate of ALS, achieving complete case confirmation and systematically collecting detailed genotype and phenotype data on ALS-related diseases within large population cohorts has been challenging.

On the other hand, due to our focus on the relationship between the retina and ALS, we have been collecting data and establishing our own clinical cohort (which we will refer to as the validation cohort), which is still undergoing recruitment. We have compiled the currently available data for further analysis: In cohort from two centers——the First Affiliated Hospital of Wenzhou Medical University and Zhejiang Provincial People's Hospital, patients with ALS were prospectively included to undergo OCT scan. The diagnosis of ALS was based on the El Escorial Criteria. The study protocol was approved by the Ethics Committee of the First Affiliated Hospital of Wenzhou Medical and Zhejiang Provincial People's Hospital, and performed in accordance with the Helsinki Declaration. All patients gave written informed consent.

As of March 2025, a total of 32 patients with ALS have been included. Additionally, 64 propensity score-matched (PSM) healthy control subjects (1:2 case-control match) were randomly selected from the hospital based on sex, age, body mass index, and smoking status.

Baseline characteristics of the cohort, compared between ALS patients and healthy controls were summarized below:

Baseline characteristics of the validation cohort.

	**ALS**	**Healthy controls**	**P value**
Sample size	32	64	
Female, n (%)	12 (37.5)	24 (37.5)	1.000
Age, years	58.38 (11.04)	55.97 (12.26)	0.141
BMI, kg/m2	22.26 (4.97)	23.46 (2.90)	0.052
Smoke, yes, n (%)	15 (46.9)	30 (46.9)	1.000
Spinal onset, n (%)	21 (65.6)	/	/

Abbreviation: ALS, Amyotrophic lateral sclerosis; BMI, Body mass index.

As shown in the table and figure below, compared to healthy controls, ALS patients had a thicker RPE (30.53 ±3 .47 vs. 27.85 ± 3.57 mm, P = 0.001) and a thinner PRL (132.83 ± 7.91 vs. 137.27 ± 8.51mm, P = 0.016). Further comparisons between patients with spinal- and bulbar-onset ALS did not reveal significant differences in PRL or RPE. Besides, we observed a thinner RNFL in patients with ALS (27.31± 3.84 vs. 29.59 ± 3.08 mm, P = 0.002)

Retinal markers in the validation cohort.

	Healthy controls	ALS	P
n	64	32	
RNFL (micrometers)	29.59 (3.08)	27.31 (3.84)	**0.002**
RPE (micrometers)	27.85 (3.57)	30.53 (3.47)	**0.001**
PRL (micrometers)	137.27 (8.51)	132.83 (7.91)	**0.016**
Macular thickness (micrometers)	266.39 (22.62)	273.39 (14.22)	0.113
Disc diameter (micrometers)	125.96 (8.59)	126.34 (11.61)	0.854
VCDR (ratio)	0.34 (0.20)	0.37 (0.18)	0.489
IPL (micrometers)	72.53 (4.77)	73.25 (5.73)	0.517
INL (micrometers)	30.44 (3.35)	31.58 (3.45)	0.123

	ALS-spinal onset	ALS-bulbar onset	P
n	21	11	
RNFL (micrometers)	27.42 (4.22)	27.10 (3.17)	0.828
RPE (micrometers)	29.85 (3.07)	31.83 (3.95)	0.126
PRL (micrometers)	134.67 (7.95)	129.33 (6.86)	0.069
Macular thickness (micrometers)	273.60 (14.51)	272.98 (14.34)	0.909
Disc diameter (micrometers)	126.55 (11.94)	125.95 (11.49)	0.891
VCDR (ratio)	0.34 (0.18)	0.42 (0.19)	0.245
IPL (micrometers)	73.66 (5.68)	72.48 (6.02)	0.588
INL (micrometers)	31.77 (3.56)	31.22 (3.35)	0.675

Abbreviation: ALS, Amyotrophic lateral sclerosis; RNFL, Retinal nerve fiber layer; IPL, inner plexiform layer; INL, Inner nuclear layer; PRL, Photoreceptor layer; RPE, Retinal pigment epithelium; VCDR, Vertical cup-to-disc ratio.

Retinal morphology in patients with amyotrophic lateral sclerosis and healthy controls.

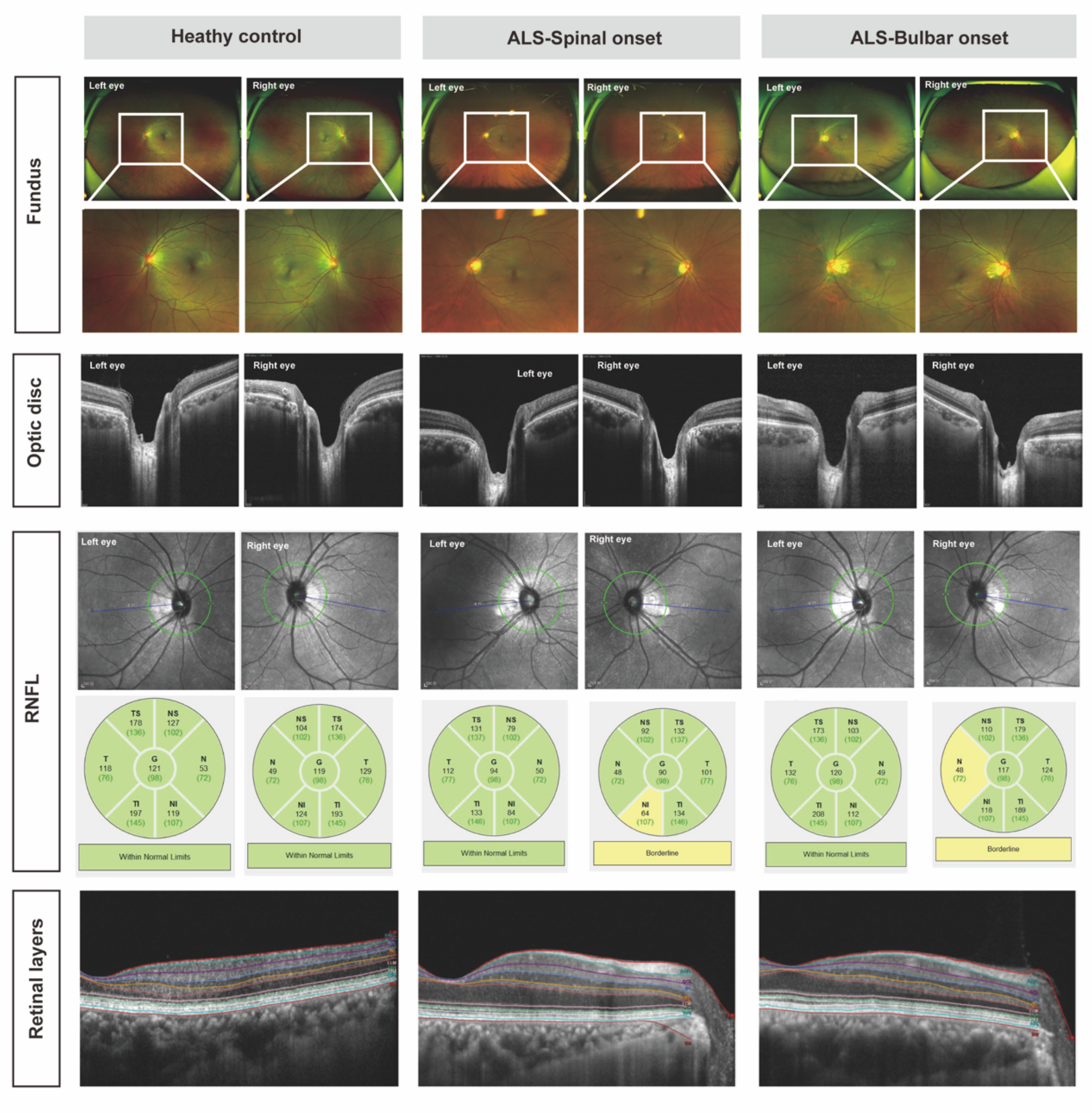

Abbreviation: ALS, Amyotrophic lateral sclerosis; RNFL, Retinal nerve fiber layer.

Analysis C: PRL, RPE and time to ALS diagnosis.

Since our study only incorporated OCT data from a single time point, we were unable to capture the dynamic changes over time. Among the 94 individuals who were ALS-free at baseline and had available OCT data, we plotted Kaplan-Meier curves for the time from study enrollment to ALS diagnosis. As shown in the figure, the low PRL group and the high RPE group had curves that were closer to the x-axis, suggesting a potentially shorter time to ALS diagnosis in these groups. However, the log-rank test indicated that these trends were not statistically significant (PRL group log-rank P=0.45; RPE group log-rank P=0.27). This may be due to the small sample size, the heterogeneity of ALS, and diagnostic delays. Future studies are needed to evaluate the longitudinal changes in ophthalmic biomarkers and their relationship with ALS progression.

Kaplan-Meier curve showing the time from OCT assessment to ALS diagnosis in patients with ALS.

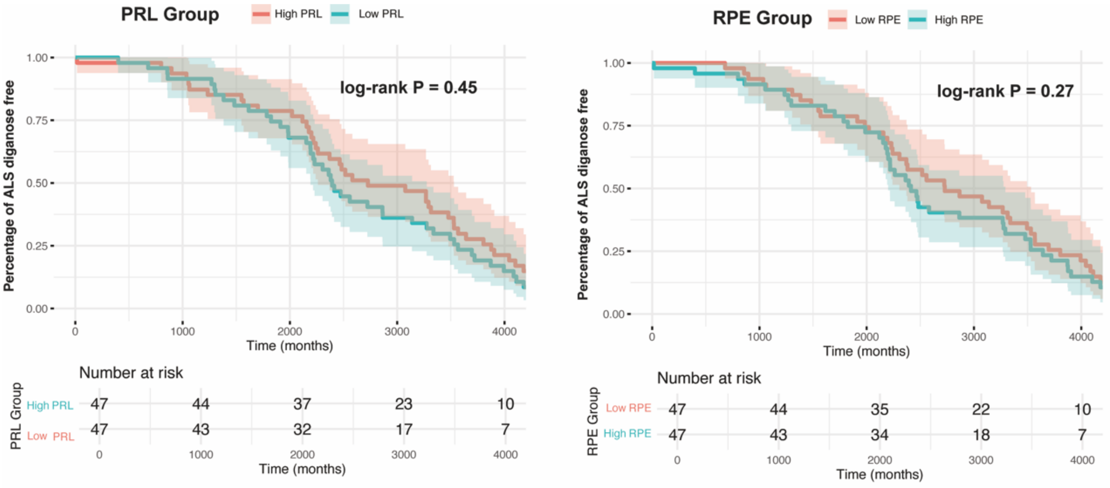

Abbreviation: ALS, Amyotrophic lateral sclerosis; OCT, Optical coherence tomography; PRL, Photoreceptor layer; RPE, Retinal pigment epithelium.

Analysis D: OCT parameters and brain MR images.

The brain MRI measurements in the UK Biobank were conducted at different time points, specifically in instances 2 and 3 (from 2014 onward and from 2019 onward, respectively), whereas the initial OCT measurements were collected in instance 0 (between 2006 and 2010). Given that structural changes in both brain MRI and OCT parameters are generally gradual and progressive and the unique value of these datasets, we conducted a correlation analysis between PRL, RPE, and selected brain MRI parameters to explore potential associations. As shown in the table below, after applying Bonferroni correction for multiple testing, PRL remained significantly positively associated with the volume of grey matter in the precentral gyrus on both sides (left: $r = 0.054$, $P < 0.001$; right: $r = 0.062$, $P < 0.001$; left and right mean: $r = 0.061$, $P < 0.001$). Notably, grey matter atrophy in the precentral gyrus has been previously implicated in ALS(1, 2), suggesting that PRL may be linked to the cortical motor neurons corresponding to this region. However, no such associations were observed with RPE.

Correlation between PRL, RPE, and Brain MRI Parameters.

	PRL		RPE	
Variable	r	P	r	P
Volume.of.grey.matter.in.Precentral.Gyrus..left	**0.054**	**0.000***	0.014	0.276
Volume.of.grey.matter.in.Precentral.Gyrus..right	**0.062**	**0.000***	0.018	0.165
Volume.of.grey.matter.in.Precentral.Gyrus..mean	**0.061**	**0.000***	0.017	0.176
Mean.FA.in.corticospinal.tract.on.FA.skeleton..left	0.028	0.031	0.023	0.070
Mean.FA.in.corticospinal.tract.on.FA.skeleton..right	0.039	0.003	0.036	0.006
Mean.FA.in.corticospinal.tract.on.FA.skeleton..mean	0.036	0.005	0.032	0.014
Mean.ICVF.in.corticospinal.tract.on.FA.skeleton..left	0.006	0.635	0.032	0.012
Mean.ICVF.in.corticospinal.tract.on.FA.skeleton..right	0.009	0.495	0.031	0.015
Mean.ICVF.in.corticospinal.tract.on.FA.skeleton..mean	0.008	0.543	0.034	0.009
Mean.ISOVF.in.corticospinal.tract.on.FA.skeleton..left	-0.006	0.651	-0.013	0.333
Mean.ISOVF.in.corticospinal.tract.on.FA.skeleton..right	-0.017	0.181	-0.01	0.462
Mean.ISOVF.in.corticospinal.tract.on.FA.skeleton..mean	-0.013	0.303	-0.013	0.320
Mean.L1.in.corticospinal.tract.on.FA.skeleton..left	0.002	0.901	-0.009	0.478
Mean.L1.in.corticospinal.tract.on.FA.skeleton..right	-0.006	0.617	-0.008	0.543
Mean.L1.in.corticospinal.tract.on.FA.skeleton..mean	-0.003	0.809	-0.009	0.504
Mean.L2.in.corticospinal.tract.on.FA.skeleton..left	-0.018	0.155	-0.019	0.145
Mean.L2.in.corticospinal.tract.on.FA.skeleton..right	-0.028	0.028	-0.022	0.087
Mean.L2.in.corticospinal.tract.on.FA.skeleton..mean	-0.025	0.049	-0.023	0.070
Mean.L3.in.corticospinal.tract.on.FA.skeleton..left	-0.02	0.116	-0.029	0.023
Mean.L3.in.corticospinal.tract.on.FA.skeleton..right	-0.032	0.012	-0.036	0.006
Mean.L3.in.corticospinal.tract.on.FA.skeleton..mean	-0.029	0.027	-0.037	0.004
Mean.MD.in.corticospinal.tract.on.FA.skeleton..left	-0.012	0.366	-0.019	0.135
Mean.MD.in.corticospinal.tract.on.FA.skeleton..right	-0.023	0.079	-0.022	0.091
Mean.MD.in.corticospinal.tract.on.FA.skeleton..mean	-0.020	0.131	-0.023	0.075
Mean.MO.in.corticospinal.tract.on.FA.skeleton..left	0.013	0.307	-0.013	0.306
Mean.MO.in.corticospinal.tract.on.FA.skeleton..right	0.005	0.72	-0.011	0.385
Mean.MO.in.corticospinal.tract.on.FA.skeleton..mean	0.009	0.503	-0.012	0.353
Mean.OD.in.corticospinal.tract.on.FA.skeleton..left	-0.011	0.402	-0.019	0.143

Mean.OD.in.corticospinal.tract.on.FA.skeleton..right	-0.022	0.095	-0.022	0.090
Mean.OD.in.corticospinal.tract.on.FA.skeleton..mean	-0.02	0.124	-0.023	0.074
Weighted.mean.FA.in.tract.corticospinal.tract..left	0.024	0.064	-0.013	0.315
Weighted.mean.FA.in.tract.corticospinal.tract..right	0.036	0.005	-0.003	0.817
Weighted.mean.FA.in.tract.corticospinal.tract..mean	0.031	0.016	-0.008	0.542
Weighted.mean.ICVF.in.tract.corticospinal.tract..left	0.003	0.799	0.023	0.080
Weighted.mean.ICVF.in.tract.corticospinal.tract..right	0.009	0.498	0.030	0.020
Weighted.mean.ICVF.in.tract.corticospinal.tract..mean	0.006	0.626	0.027	0.036
Weighted.mean.ISOVF.in.tract.corticospinal.tract..left	-0.017	0.179	-0.001	0.94
Weighted.mean.ISOVF.in.tract.corticospinal.tract..right	-0.022	0.083	-0.003	0.824
Weighted.mean.ISOVF.in.tract.corticospinal.tract..mean	-0.022	0.092	-0.004	0.778
Weighted.mean.L1.in.tract.corticospinal.tract..left	0.006	0.615	-0.023	0.078
Weighted.mean.L1.in.tract.corticospinal.tract..right	0.006	0.618	-0.017	0.186
Weighted.mean.L1.in.tract.corticospinal.tract..mean	0.006	0.623	-0.022	0.092
Weighted.mean.L2.in.tract.corticospinal.tract..left	-0.018	0.161	0.005	0.714
Weighted.mean.L2.in.tract.corticospinal.tract..right	-0.033	0.012	-0.003	0.841
Weighted.mean.L2.in.tract.corticospinal.tract..mean	-0.026	0.041	0.000	0.998
Weighted.mean.L3.in.tract.corticospinal.tract..left	-0.028	0.028	-0.006	0.617
Weighted.mean.L3.in.tract.corticospinal.tract..right	-0.036	0.005	-0.019	0.134
Weighted.mean.L3.in.tract.corticospinal.tract..mean	-0.034	0.01	-0.014	0.284
Weighted.mean.MD.in.tract.corticospinal.tract..left	-0.015	0.246	-0.012	0.351
Weighted.mean.MD.in.tract.corticospinal.tract..right	-0.022	0.09	-0.017	0.180
Weighted.mean.MD.in.tract.corticospinal.tract..mean	-0.019	0.136	-0.017	0.179
Weighted.mean.MO.in.tract.corticospinal.tract..left	0.010	0.443	-0.021	0.099
Weighted.mean.MO.in.tract.corticospinal.tract..right	0.016	0.206	-0.031	0.017
Weighted.mean.MO.in.tract.corticospinal.tract..mean	0.014	0.269	-0.028	0.028
Weighted.mean.OD.in.tract.corticospinal.tract..left	-0.032	0.014	0.034	0.008
Weighted.mean.OD.in.tract.corticospinal.tract..right	-0.033	0.01	0.023	0.079
Weighted.mean.OD.in.tract.corticospinal.tract..mean	-0.034	0.009	0.030	0.022

*Significant after Bonferroni correction

Abbreviation: PRL, Photoreceptor layer; RPE, Retinal pigment epithelium.

Analysis E: MR analysis of OCT-related parameters and ALS.

Due to the limitations of causality, we searched the genome-wide association study (GWAS) database and conducted some MR analysis:

The exposure and outcome GWAS data used in this study were obtained from an open-access database (https://gwas.mrcieu.ac.uk), the study employed two-sample MR analysis to assess the causal relationship between OCT-related parameters and ALS. MR is a method that uses genetic instruments to study causal relationships between modifiable exposures and outcomes. We employed three different MR methods for analysis: MR-Egger, Weighted Median, and the Inverse Variance Weighted method. GWAS data for ALS were derived from a cohort consisting of 138,086 individuals of European ancestry[44]. As for OCT-related parameters, only RNFL and GCIPL were available. Meanwhile, due to the lack of GWAS data specifically for PRL, RPE, and many other OCT parameters, we included certain ophthalmic diseases associated with these parameters as proxies to infer potential associations.

As detailed in the table below, our analysis did not reveal a significant causal relationship between RNFL (MR-Egger OR = 1.018, P = 0.633) or GCIPL (MR-Egger OR = 1.000, P = 0.997) and ALS, a finding consistent with certain aspects of our study results. Furthermore, no evidence of causality was observed between age-related macular degeneration, other disorders of the choroid, and retinoschisis and retinal cysts:

MR results of OCT-related parameters and ALS.

Exposure (GWAS ID)	Method	nsnp	OR	0R_lci95	OR_uci95	P
RNFL<br>(ebi-a-GCST90014266)	MR Egger	21	1.018	0.948	1.093	0.633
	Weighted median	21	0.999	0.971	1.027	0.929
	Inverse variance weighted	21	0.998	0.974	1.022	0.852
GCIPL<br>(bi-a-GCST90014267)	MR Egger	18	1.000	0.953	1.049	0.997
	Weighted median	18	0.991	0.971	1.011	0.377
	Inverse variance weighted	18	0.993	0.976	1.010	0.423
Age-related macular degeneration<br>(finn-b-H7_AMD)	MR Egger	6	0.983	0.933	1.036	0.557
	Weighted median	6	0.995	0.972	1.019	0.686
	Inverse variance weighted	6	0.998	0.974	1.022	0.852
Other disorders of choroid<br>(finn-b-H7_CHOROIDOTH)	MR Egger	4	0.891	0.783	1.014	0.222
	Weighted median	4	0.958	0.866	1.060	0.407
	Inverse variance weighted	4	0.997	0.891	1.116	0.964
Retinoschisis and retinal cysts<br>(finn-b-H7_RETINOCHISISCYST)	MR Egger	6	1.039	0.860	1.254	0.713
	Weighted median	6	1.066	0.931	1.220	0.356
	Inverse variance weighted	6	0.999	0.900	1.109	0.986

Abbreviation: ALS, Amyotrophic lateral sclerosis; OCT, Optical coherence tomography; MR, Mendelian analysis.

Analysis F: MR analysis of retinal vascular features and ALS.

The retinal vasculature can be assessed using non-invasive imaging techniques, and its visualization has also been explored in the evaluation of neurodegenerative diseases such as Alzheimer's disease and Parkinson's disease[54,55]. Changes in the small blood vessels of the skin, muscles, and brain have been reported in ALS patients[56,57], indicating the rationale for considering retinal microvasculature as a potential marker for this disease. Abnormal retinal vascular formation has been explored in ALS, with Abdelhak et al. [29]reporting a significant increase in the outer wall thickness of retinal vessels, while another study using optical coherence tomography angiography found no ALS-specific alterations[41]. Nevertheless, to date, research on retinal vasculature in ALS remains a relatively novel field with limited evidence, highlighting the need for further studies to substantiate these findings.

However, in the UK Biobank, there is no data on retinal vasculature. Therefore, we utilized GWAS data related to retinal vasculature to perform a MR analysis, exploring the association between retinal vascular factors and ALS risk： The exposure and outcome GWAS data used in this study were obtained from an open-access database (https://gwas.mrcieu.ac.uk), the study employed two-sample MR analysis to assess the causal relationship between retinal vasculature parameters and ALS. MR is a method that uses genetic instruments to study causal relationships between modifiable exposures and outcomes. We employed three different MR methods for analysis: MR-Egger, Weighted Median, and the Inverse Variance Weighted method. GWAS data for ALS were derived from a cohort consisting of 138,086 individuals of European ancestry[44].

As shown in the table, no significant genetic correlation with ALS was found for retinal vascular occlusion, retinal vein occlusion, retinal hemorrhage, or background retinopathy and retinal vascular changes：

Mendelian randomization analysis between retinal vascular factors and ALS.

Exposure (GWAS ID)	Method	nsnp	or	or_lci95	or_uci95	P
Retinal vascular occlusion	MR Egger	5	1.332	0.294	6.025	0.735
(finn-b-H7_RETIVASCOCCLUSION)	Weighted median	5	0.964	0.012	75.759	0.987
	Inverse variance weighted	5	0.919	0.690	1.224	0.562
Retinal vein occlusion	MR Egger	3	1.046	0.828	1.321	0.770
(finn-b-DM_RET_VEIN_OCCLU)	Weighted median	3	1.016	0.826	1.249	0.881
	Inverse variance weighted	3	0.966	0.815	1.145	0.689
Retinal hemorrhage	MR Egger	5	0.868	0.674	1.119	0.355
(finn-b-H7_RETINAHAEMORR)	Weighted median	5	0.927	0.765	1.123	0.438
	Inverse variance weighted	5	1.042	0.908	1.196	0.555
Background retinopathy and retinal	MR Egger	3	0.941	0.660	1.343	0.795
vascular changes	Weighted median	3	1.029	0.815	1.300	0.807
(finn-b-H7_BCKRNDRETINOPAT)	Inverse variance weighted	3	1.134	0.991	1.299	0.068

Abbreviation: ALS, Amyotrophic lateral sclerosis; OCT, Optical coherence tomography; MR, Mendelian analysis.

Analysis G: Retinal microvascular Changes and ALS.

Validation cohort: due to our focus on the relationship between the retina and ALS, we have been collecting data and establishing our own clinical cohort (which we will refer to as the validation cohort), which is still undergoing recruitment. We have compiled the currently available data for further analysis: In cohort from two centers——the First Affiliated Hospital of Wenzhou Medical University and Zhejiang Provincial People's Hospital, patients with ALS were prospectively included to undergo optical coherence tomography angiography testing. The diagnosis of ALS was based on the El Escorial Criteria. The study protocol was approved by the Ethics Committee of the First Affiliated Hospital of Wenzhou Medical and Zhejiang Provincial People's Hospital, and performed in accordance with the Helsinki Declaration. All patients gave written informed consent.

As of March 2025, a total of 32 patients with ALS have been included. Additionally, 64 propensity score-matched (PSM) healthy control subjects (1:2 case-control match) were randomly selected from the hospital based on sex, age, body mass index, and smoking status.

Baseline characteristics of the cohort, compared between ALS patients and healthy controls were summarized below:

Baseline characteristics of the validation cohort.

	**ALS**	**Healthy controls**	**P value**
Sample size	32	64	
Female, n (%)	12 (37.5)	24 (37.5)	1.000
Age, years	58.38 (11.04)	55.97 (12.26)	0.141
BMI, kg/m2	22.26 (4.97)	23.46 (2.90)	0.052
Smoke, yes, n (%)	15 (46.9)	30 (46.9)	1.000
Spinal onset, n (%)	21 (65.6)	/	/

Abbreviation: ALS, Amyotrophic lateral sclerosis; BMI, Body mass index.

The microvascular density was calculated for the 2.5-mm-diameter total annular zone (TAZ) after excluding the foveal avascular zone (diameter 1⁄4 0.6 mm).The methods above were implemented using MATLAB v7.10 (MathWorks, Inc., Natick, MA, USA). As shown in the table and figure below, no significant differences between the superficial and deep vasculature were found among healthy controls and ALS patients.

Comparison of the microvascular density between the ALS patients and healthy controls

	Healthy controls	ALS	P
n	64	32	
SRCP	60. 76 ±4.21	59.66± 4.10	0.531
DRCP	69.76 ± 5.39	68.92± 5.79	0.446
Whole retinal capillary plexus	62.51 ± 4.78	61.92± 4.94	0.103
	ALS-spinal onset	ALS-bulbar onset	P
n	21	11	
SRCP	61.20 ±5.72	60.96± 5.57	0.829
DRCP	68.16 ± 5.46	67.81± 4.98	0.730
Whole retinal capillary plexus	62.28 ± 4.79	61.49± 5.39	0.229

Abbreviation: SRCP, superficial retinal capillary plexus; DRCP, deep retinal capillary plexus.

Representative OCT-A images between the ALS patients and healthy controls.

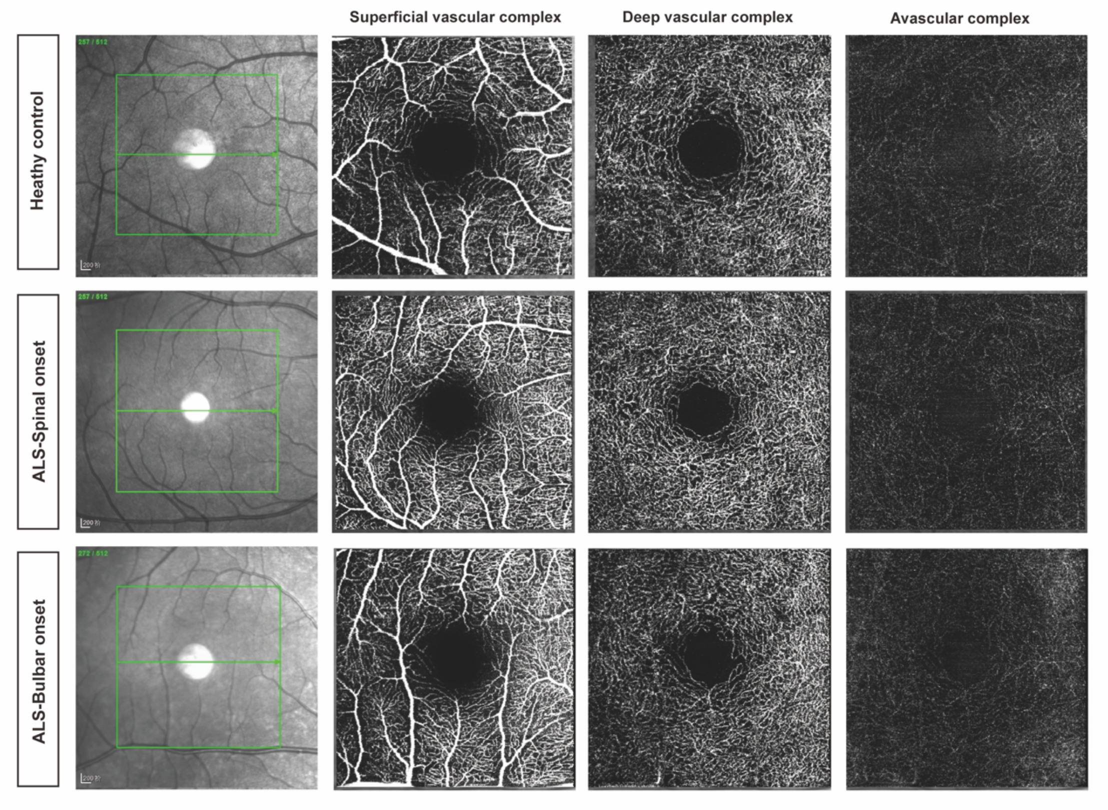

In the image, the ALS group shows a thinner RNFL compared to the healthy group, but no significant abnormalities are observed in either the large vessels or microvasculature. In summary, results from our validation cohort did not identify any specificity of retinal vascular indicators in relation to ALS. However, our cohort is still in the process of recruitment, and we plan to further expand the sample size to investigate the relationships between retinal vascular indicators and disease phenotypes, genotypes, and survival prognosis in the future. Overall, this study currently lacks evidence regarding the association between retinal vascular and ALS.

References

1. Wagner SK, Romero-Bascones D, Cortina-Borja M, Williamson DJ, Struyven RR, Zhou Y, et al. Retinal Optical Coherence Tomography Features Associated With Incident and Prevalent Parkinson Disease. Neurology. 2023;101(16):e1581-e93.

2. Ishaque A, Ta D, Khan M, Zinman L, Korngut L, Genge A, et al. Distinct patterns of progressive gray and white matter degeneration in amyotrophic lateral sclerosis. Hum Brain Mapp. 2022;43(5):1519-34.

3. van Rheenen W, van der Spek RAA, Bakker MK, van Vugt J, Hop PJ, Zwamborn RAJ, et al. Common and rare variant association analyses in amyotrophic lateral sclerosis identify 15 risk loci with distinct genetic architectures and neuron-specific biology. Nat Genet. 2021;53(12):1636-48.

4. Cabrera DeBuc D, Somfai GM, Koller A. Retinal microvascular network alterations: potential biomarkers of cerebrovascular and neural diseases. Am J Physiol Heart Circ Physiol. 2017;312(2):H201-h12.

5. Liew G, Wang JJ, Mitchell P, Wong TY. Retinal vascular imaging: a new tool in microvascular disease research. Circ Cardiovasc Imaging. 2008;1(2):156-61.

6. Buckley AF, Bossen EH. Skeletal muscle microvasculature in the diagnosis of neuromuscular disease. J Neuropathol Exp Neurol. 2013;72(10):906-18.

7. Kolde G, Bachus R, Ludolph AC. Skin involvement in amyotrophic lateral sclerosis. Lancet. 1996;347(9010):1226-7.

8. Abdelhak A, Hübers A, Böhm K, Ludolph AC, Kassubek J, Pinkhardt EH. In vivo assessment of retinal vessel pathology in amyotrophic lateral sclerosis. J Neurol. 2018;265(4):949-53.

9. Cennamo G, Montorio D, Ausiello FP, Magno L, Iodice R, Mazzucco A, et al. Correlation between Retinal Vascularization and Disease Aggressiveness in Amyotrophic Lateral Sclerosis. Biomedicines. 2022;10(10).
