# Supplementary material for: Analysis of retinal markers and incident amyotrophic lateral sclerosis: An optical coherence tomography-based cohort study
Source: PLoS Med. 2025 Jun 25;22(6):e1004545. doi: 10.1371/journal.pmed.1004545 (PMC12193630; doi:10.1371/journal.pmed.1004545)
Supplement: Attachment — Submitted filename: Respond to General editorial request.pdf [file pmed.1004545.s005.pdf]

Respond to General editorial request

Comment* *We ask every co-author listed on the manuscript to fill in a contributing author statement, making sure to declare all competing interests. If any of the co-authors have not filled in the statement, we will remind them to do so when the paper is revised. If all statements are not completed in a timely fashion this could hold up the re-review process. If new competing interests are declared later in the revision process, this may also hold up the submission. Should there be a problem getting one of your co-authors to fill in a statement we will be in contact. Please do not add or remove authors without first discussing this with the handling editor. You can see our competing interests policy here: http://journals.plos.org/plosmedicine/s/competing-interests.*

Response: Thank you for your feedback. All authors have already filled in a Contributing Author Statement, ensuring that all competing interests are declared.

Comment* *Please upload any figures associated with your paper as individual TIF or EPS files with 300dpi resolution at resubmission; please read our figure guidelines for more information on our requirements: http://journals.plos.org/plosmedicine/s/figures. While revising your submission, please upload your figure files to the PACE digital diagnostic tool, https://pacev2.apexcovantage.com/. PACE helps ensure that figures meet PLOS requirements. To use PACE, you must first register as a user. Then, login and navigate to the UPLOAD tab, where you will find detailed instructions on how to use the tool. If you encounter any issues or have any questions when using PACE, please email us at PLOSMedicine@plos.org.*

Response: Thank you for the guidance. We have checked our figures, and they meet the required specifications. If any further adjustments are needed, please let us know.

Comment* *please ensure that the paper adheres to the PLOS Data Availability Policy (see http://journals.plos.org/plosmedicine/s/data-availability), which requires that all data underlying the study's findings be provided in a repository or as Supporting Information. For data residing with a third party, authors are required to provide instructions with contact information (web or email address) for obtaining the data. Please note that a study author cannot be the contact person for the data. PLOS journals do not allow statements supported by "data not shown" or "unpublished results." For such statements, authors must provide supporting data or cite public sources that include it.*

Response: Thank you for your guidance. The following statement has already been included in the manuscript:

Acknowledgement sections (Page 8-9, Line 350-353): :The data supporting the findings of this study are available on the UK Biobank project site and are subject to successful registration and application processes. Further details are available at https://www. UK biobank. ac=/. Our research data are available upon reasonable request.

Comment* *We expect all researchers with submissions to PLOS in which author-generated code underpins the findings in the manuscript to make all author-generated code available without restrictions upon publication of the work. In cases where code is central to the manuscript, we may require the code to be made available as a condition of publication. Authors are responsible for ensuring that the code is reusable and well documented. Please make any custom code available, either as part of your data deposition or as a supplementary file. Please add a sentence to your data*

availability statement regarding any code used in the study, e.g. "The code used in the analysis is available from Github [URL] and archived in Zenodo [DOI link]" Please review our guidelines at https://journals.plos.org/plosmedicine/s/materials-software-and-code-sharing and ensure that your code is shared in a way that follows best practice and facilitates reproducibility and reuse. Because Github depositions can be readily changed or deleted, we encourage you to make a permanent DOI'd copy (e.g. in Zenodo) and provide the URL.

Response: Thank you for your guidance. Our study did not use any custom code that underpins the findings. If any further clarification is needed, please let us know.

Comment* *FINANCIAL DISCLOSURES: The funding statement should include: specific grant numbers, initials of authors who received each award, URLs to sponsors' websites. Also, please state whether any sponsors or funders (other than the named authors) played any role in study design, data collection and analysis, the decision to publish, or preparation of the manuscript. If they had no role in the research, include this sentence: "The funders had no role in study design, data collection and analysis, decision to publish, or preparation of the manuscript."*

Response: Thank you for your guidance. The funding statement has been included in the manuscript as follows:

Acknowledgement sections (Page 9, Line 357-359): This research was supported by the National Natural Science Foundation of China (81901273 to Binbin Deng) and the Natural Science Foundation of Zhejiang Province (ZCLY24H0903 to Binbin Deng). Professor Binbin Deng provided resources and designed the study.

Comment* *COMPETING INTERESTS: All authors must declare their relevant competing interests per the PLOS policy, which can be seen here: https://journals.plos.org/plosmedicine/s/competing-interests*

Response: Thank you for your guidance. The funding statement has been included in the manuscript as follows:

Acknowledgement sections (Page 9, Line 355): The authors have stated explicitly that there are no conflicts of interest in connection with this article.

Comment* *DATA AVAILABILITY: The Data Availability Statement (DAS) requires revision. PLOS Medicine requires that the de-identified data underlying the specific results in a published article be made available, without restrictions on access, in a public repository or as Supporting Information at the time of article publication, provided it is legal and ethical to do so. Please see the policy at http://journals.plos.org/plosmedicine/s/data-availability and FAQs at http://journals.plos.org/plosmedicine/s/data-availability#loc-faqs-for-data-policy*

Response: Thank you for your guidance. The following statement has already been included in the manuscript:

Acknowledgement sections (Page 8-9, Line 350-353) :The data supporting the findings of this study are available on the UK Biobank project site and are subject to successful registration and application processes. Further details are available at https://www. UK biobank. ac=/. Our research data are available upon reasonable request.

FORMATTING - GENERAL

Comment* *Abstract: Please structure your abstract using the PLOS Medicine headings (Background, Methods and Findings, Conclusions). Please combine the Methods and Findings sections into one section.*

Response: Thank you for your guidance. The abstract has been revised according to the PLOS Medicine guidelines：

Abstract sections (Page 1, Line 25-42):

Background: Biomarkers are widely recognized as crucial breakthroughs in tackling amyotrophic lateral sclerosis (ALS). Among them, retina markers may hold promise due to the close retina-brain connection and non-invasive, portable detection methods. Thus, using optical coherence tomography (OCT), we investigated the link between baseline cell-level retinal features and future ALS risk.

Methods and Findings: Participants from the UK Biobank underwent OCT scans to assess retinal layers, macula, and optic disc parameters. Follow-up began 2 years after baseline (year 2006-1010), with ALS cases identified through ICD codes from medical and assessment records. Cox proportional hazards models were applied to examine the prospective relationship between retinal markers and incident ALS. Over a median follow-up of 14.11 years, the ALS incidence was 10.58 per 100,000 person-years among 53,824 participants. After adjusting for covariates, per standard deviation (SD) decrease of 15.19 mm in photoreceptor layer thickness was strongly associated with a 19% increased risk of ALS (HR [95% CI] = 0.81 [0.71–0.93], P = 0.002), while per SD increase of 26.11 mm in retinal pigment epithelium corresponded to a 20% higher risk (HR [95% CI] = 1.20 [1.07–1.34], P = 0.002). Sensitivity analyses excluding follow-ups of less than 4 and 6 years yielded consistent results. Subgroup analyses showed these findings were more pronounced in smokers.

Conclusion: A thinner photoreceptor layer and thicker retinal pigment epithelium may precede the clinical diagnosis of ALS, offering potential clues for early diagnosis and insights into the disease's pathogenesis.

Comment* *At this stage, we ask that you include a short, non-technical Author Summary of your research to make findings accessible to a wide audience that includes both scientists and non-scientists. The Author Summary should immediately follow the Abstract in your revised manuscript. This text is subject to editorial change and should be distinct from the scientific abstract. Ideally each sub-heading should contain 2-3 single sentence, concise bullet points containing the most salient points from your study. In the final bullet point of 'What Do These Findings Mean?', please include the main limitations of the study in non-technical language. Please see our author guidelines for more information: https://journals.plos.org/plosmedicine/s/revising-your-manuscript#loc-author-summary.*

Response: Thank you for your feedback. A short, non-technical Author Summary has been added to the manuscript immediately following the abstract, as per your request：

Author Summary (Page 2, Line 45-56):

- **Why Was This Study Done?** Retinal biomarkers are gaining recognition for amyotrophic lateral sclerosis (ALS) diagnosis due to the retina-brain connection and non-invasive detection techniques, though evidence of early retinal alterations in ALS remains limited.

- **What Did the Researchers Do and Find?** Based on optical coherence tomography, among 53,824 participants, the relationship between cell-level retinal features and future ALS risk

was investigated over a median follow-up of 14.11 years. We found that a thinner photoreceptor layer and a thicker retinal pigment epithelium may precede the clinical diagnosis of ALS.

- **What Do These Findings Mean?** Assessment of the photoreceptor layer and retinal pigment epithelium thickness by optical coherence tomography may offer potential clues for early diagnosis and provide insights into the pathogenesis of ALS.

Comment* *Please express the main results with 95% CIs as well as p values. When reporting p values please report as p<0.001 and where higher as the exact p value p=0.002, for example. Throughout, suggest reporting statistical information as follows to improve clarity for the reader "22% (95% CI [13%,28%]; p</=)". Please be sure to define all numerical values at first use.*

Response: Thank you for your guidance. The manuscript has been updated to express the main results with 95% confidence intervals (CIs) and p-values as requested.

Comment* *Please include page numbers and line numbers in the manuscript file. Use continuous line numbers (do not restart the numbering on each page).*

Response: Thank you for your guidance. Page numbers and continuous line numbers have been added to the manuscript as requested. Please let us know if any further adjustments are needed.

Comment* *Please cite the reference numbers in square brackets. Citations should precede punctuation.*

Response: Thank you for your guidance. The manuscript has been thoroughly checked, and all references have been cited in square brackets, with citations preceding punctuation as required. Please let us know if any further revisions are necessary.

FIGURES AND TABLES

Comment* *Please provide titles and legends for all figures and tables (including those in Supporting Information files).*

Response: Thank you for your feedback. Titles and legends have been added to all figures and tables, including those in the Supporting Information files, as requested. Please let us know if any further revisions are needed.

Comment* *Please define all abbreviations used in each figure/table (including those in Supporting Information files).*

Response: Thank you for the feedback. The manuscript has been carefully reviewed, and all abbreviations used in each figure and table, including those in the Supporting Information files, have been defined. Please let me know if any further adjustments are necessary.

Comment* *Please consider avoiding the use of red and green in order to make your figure more accessible to those with color blindness.*
Response: Thank you for the suggestion. Red and green have not been used to distinguish content in the figures.

SUPPLEMENTARY MATERIAL
Comment* *Please note that supplementary material will be posted as supplied by the authors. Therefore, please amend it according to the relevant comments outlined here.*
Response: Thank you for your feedback. The supplementary material has been amended according to the relevant comments as outlined.

Comment* *Please cite your Supporting Information as outlined here: https://journals.plos.org/plosmedicine/s/supporting-information*
Response: Thank you for your feedback. The Supporting Information citations have been checked and are in compliance with the guidelines. Please let me know if any further revisions are needed.

REFERENCES
Comment* *PLOS uses the numbered citation (citation-sequence) method and first six authors, et al.*
Response: Thank you for your feedback. The references have been revised to follow the PLOS citation-sequence method, with the first six authors listed followed by "et al." as required. Please let me know if any further adjustments are necessary.

Comment* *Please ensure that journal name abbreviations match those found in the National Center for Biotechnology Information (NCBI) databases (http://www.ncbi.nlm.nih.gov/nlmcatalog/journals), and are appropriately formatted and capitalised.*
Response: Thank you for your feedback. The journal name abbreviations have been checked and are consistent with those found in the National Center for Biotechnology Information (NCBI) databases. They are appropriately formatted and capitalized. Please let me know if any further revisions are needed.

Comment* *Where website addresses are cited, please include the complete URL and specify the date of access (e.g. [accessed: 12/06/2023]).*
Response: Thank you for your feedback. No websites have been cited in the manuscript. Please let me know if any further adjustments are needed.
Comment* *Please also see https://journals.plos.org/plosmedicine/s/submission-guidelines#loc-references for further details on reference formatting.*
Response: Thank you for your feedback. We have checked the format of the references.

STUDY TYPE-SPECIFIC REQUESTS

Comment* *Abstract: Please include the study design, population and setting, number of participants, years during which the study took place (enrollment and follow up), length of follow up, and main outcome measures.*

Response: Thank you for your guidance. The required details have been included in the abstract, as indicated by the red markings. Please let me know if any further revisions are needed.

Abstract sections (Page 1, Line 25-42):

Background: Biomarkers are widely recognized as crucial breakthroughs in tackling amyotrophic lateral sclerosis (ALS). Among them, retina markers may hold promise due to the close retina-brain connection and non-invasive, portable detection methods. Thus, using optical coherence tomography (OCT), we investigated the link between baseline cell-level retinal features and future ALS risk.

Methods and Findings: Participants from the UK Biobank underwent OCT scans to assess retinal layers, macula, and optic disc parameters. Follow-up began 2 years after baseline (year 2006-1010), with ALS cases identified through ICD codes from medical and assessment records. Cox proportional hazards models were applied to examine the prospective relationship between retinal markers and incident ALS. Over a median follow-up of 14.11 years, the ALS incidence was 10.58 per 100,000 person-years among 53,824 participants. After adjusting for covariates, per standard deviation (SD) decrease of 15.19 mm in photoreceptor layer thickness was strongly associated with a 19% increased risk of ALS (HR [95% CI] = 0.81 [0.71–0.93], P = 0.002), while per SD increase of 26.11 mm in retinal pigment epithelium corresponded to a 20% higher risk (HR [95% CI] = 1.20 [1.07–1.34], P = 0.002). Sensitivity analyses excluding follow-ups of less than 4 and 6 years yielded consistent results. Subgroup analyses showed these findings were more pronounced in smokers.

Conclusion: A thinner photoreceptor layer and thicker retinal pigment epithelium may precede the clinical diagnosis of ALS, offering potential clues for early diagnosis and insights into the disease's pathogenesis.

Comment* *Please ensure that the study is reported according to the STROBE (or appropriate STOBE extension) guideline (available from: https://www.equator-network.org/reporting-guidelines/strobe) and include the completed STROBE (or STROBE extension) checklist as Supporting Information. Please add the following statement, or similar, to the Methods: "This study is reported as per the Strengthening the Reporting of Observational Studies in Epidemiology (STROBE) guideline (S1 Checklist)." When completing the checklist, please use section and paragraph numbers, rather than page numbers.*

Response: The necessary modifications have been made, and the corresponding content has been added:

Method Sections (Page 3, line 115-117): This study is reported as per the Strengthening the Reporting of Observational Studies in Epidemiology (STROBE) guideline (S1 Checklist).

Comment* *For all observational studies, in the manuscript text, please indicate: (1) the specific hypotheses you intended to test, (2) the analytical methods by which you planned to test them, (3) the analyses you actually performed, and (4) when reported analyses differ from those that were planned, transparent explanations for differences that affect the reliability of the study's results. If a reported*

analysis was performed based on an interesting but unanticipated pattern in the data, please be clear that the analysis was data driven.

Response: Thanks for your feedback. We have reviewed the entire manuscript and confirmed that it includes the required content.

Comment* *Please state in the Methods section whether the study had a prospective protocol or analysis plan. If a prospective analysis plan (from your funding proposal, IRB or other ethics committee submission, study protocol, or other planning document written before analyzing the data) was used in designing the study, please include the relevant document(s) with your revised manuscript as a Supporting Information file to be published alongside your study and cite it in the Methods section. A legend for this file should be included at the end of your manuscript. If no such document exists, please make sure that the Methods section transparently describes when analyses were planned, and when/why any data-driven changes to analyses took place. Changes in the analysis, including those made in response to peer review comments, should be identified as such in the Methods section of the paper, with rationale.*

Response: Thanks for your feedback. This is a retrospective study using the UK Biobank; thus, it is not applicable.
