# Supplementary material for: Analysis of retinal markers and incident amyotrophic lateral sclerosis: An optical coherence tomography-based cohort study
Source: PLoS Med. 2025 Jun 25;22(6):e1004545. doi: 10.1371/journal.pmed.1004545 (PMC12193630; doi:10.1371/journal.pmed.1004545)
Supplement: Attachment — Submitted filename: respond-r2.pdf [file pmed.1004545.s006.pdf]


Comments from Reviewers:

Reviewer #1: Alex McConnachie, Statistical Review

I thank Pang and colleagues for their consideration of my original comments, and I am happy with their responses, and the changes to the paper.

One very minor exception is the new text at lines 178 to 182. Personally, I would remove "before standardization" since this will have no impact on skewness.

Response: Thank you very much for your kind comments and for acknowledging our revisions. We truly appreciate your insightful feedback. Following your suggestion, we have removed the phrase "before standardization" from lines 178 to 182.

Reviewer #3: I *believe the authors have addressed my and the other reviewers comments in a thoughtful and thorough manner and enhanced the quality of the manuscript*

Response: Thank you very much for your positive feedback. We truly appreciate your careful review and are grateful for your recognition of our efforts to improve the manuscript. Your comments have been invaluable in helping us enhance the overall quality and clarity of our work.

Reviewer #4: *I thank the authors for their careful and thorough revision. I found their responses clear, thoughtful, and scientifically sound. The main concerns I raised—particularly regarding causal inference, the interpretation of PRL and RPE, and the absence of vascular OCT parameters—were addressed appropriately. The inclusion of new analyses, clarification of terminology, and validation in a clinical cohort all contribute to strengthening the manuscript. I have no further major concerns and support the revised version for publication.*

Response: Thank you very much for your thoughtful and encouraging comments. We sincerely appreciate your recognition of our efforts in addressing the concerns raised. Your constructive feedback greatly contributed to improving the clarity and scientific rigor of our manuscript. We are truly grateful for your support of the revised version.

Requests from Editors:

GENERAL

Comment 1: * *Please confirm that your title complies with to PLOS Medicine's style. Your title must be nondeclarative and not a question. It should begin with main concept if possible. "Effect of" should be used only if causality can be inferred, i.e., for an RCT. Please place the study design ("A randomized controlled trial," "A retrospective study," "A modelling study," etc.) in the subtitle (i.e., after a colon). Suggestion: Analysis of retinal markers and incident amyotrophic lateral sclerosis: an optical coherence tomography-based cohort study*

Response: Thank you for the helpful guidance regarding the title format. We have revised the title according to your suggestion: Analysis of retinal markers and incident amyotrophic lateral sclerosis: an optical coherence tomography-based cohort study.

Comment 2: * *Statistical reporting: Please revise throughout the manuscript, including tables and figures.*

- Please report statistical information as follows to improve clarity for the reader ""22%(95% CI [13,28]; p</=)"".

- Please separate upper and lower bounds with commas instead of hyphens as the latter can be confused with reporting of negative values.

- Please repeat statistical definitions (HR, CI etc.) for each set of parentheses.

Response: Thank you for the suggestion. The statistical reporting has been revised throughout the manuscript, particularly in the Results section and all relevant tables and figures. Definitions such as HR and CI have been repeated for each set of parentheses, and commas have been used to separate confidence interval bounds as recommended (changes were marked in red).

Comment 3: * *Please ensure that all abbreviations are defined at first use throughout the text (including statistical abbreviations). Please also check figures and tables.*

Response: Thank you for the comment. All abbreviations, including statistical terms, have been defined at their first occurrence in the main text, as well as in all figures and tables.

Comment 4: * *Please ensure that tables and figures, including those in supplementary files, are appropriately referenced in the main text.*

Response: Thank you for the reminder. All tables and figures, including those in the supplementary

files, have been carefully checked and appropriately referenced in the main text.

Comment 5:* *Financial Disclosure: The funding statement should include: specific grant numbers, initials of authors who received each award, URLs to sponsors' websites. Also, please state whether any sponsors or funders (other than the named authors) played any role in study design, data collection and analysis, the decision to publish, or preparation of the manuscript. If they had no role in the research, include this sentence: "The funders had no role in study design, data collection and analysis, decision to publish, or preparation of the manuscript."*

Response: Thank you for your valuable comments. The financial disclosure has been updated accordingly. The current version reads:

This research was supported by the National Natural Science Foundation of China (Grant No. 81901273 to Binbin Deng, http://www.nsfc.gov.cn) and the Natural Science Foundation of Zhejiang Province (Grant No. ZCLY24H0903 to Binbin Deng, http://www.zjkjt.gov.cn). Professor Binbin Deng provided resources and designed the study. The funders had no role in study design, data collection and analysis, decision to publish, or preparation of the manuscript.

Comment 6:* *The Data Availability Statement (DAS) requires revision. For each data source used in your study:*

a) If the data are freely or publicly available, note this and state the location of the data: within the paper, in Supporting Information files, or in a public repository (include the DOI or accession number).

b) If the data are owned by a third party but freely available upon request, please note this and state the owner of the data set and contact information for data requests (web or email address). Note that a study author cannot be the contact person for the data.

c) If the data are not freely available, please describe briefly the ethical, legal, or contractual restriction that prevents you from sharing it. Please also include an appropriate contact (web or email address) for inquiries (again, this cannot be a study author).

Response: Thank you for the suggestion. The Data Availability Statement has been revised accordingly as follows:

The data supporting the findings of this study are available through the UK Biobank project site and require successful registration and application due to ethical and legal restrictions protecting participant privacy and data security. Further details on access procedures can be found at https://www.ukbiobank.ac.uk. Our research data derived from the UK Biobank dataset are available from the UK Biobank upon reasonable request. For data access inquiries, please contact the UK Biobank directly via their website or email: access@ukbiobank.ac.uk.

Comment 7: ** Please revise for use of patient-centered language. Please note that patient-centered language is constructed with the use of post-modified nouns (e.g. 'patients with ALS' (or similar) instead of 'ALS patients') putting the person first in the sentence structure.*

Response: Thank you for the valuable comment. The manuscript has been carefully reviewed, and all instances have been revised to use patient-centered language, such as "patients with ALS" instead of "ALS patients," putting the person first throughout the text.

Comment 8: ** Please ensure that changes in the analysis -- including those made in response to peer review comments -- are identified as such in the Methods section of the paper, with rationale.*

Response: Thank you for the suggestion. The Methods section has been revised as follows to clearly identify changes made in response to peer review comments, with rationale (marked in red):

Method sections (Page4-5, Line 164-172): In the post hoc analysis (Figure 1), subgroup analyses were conducted, stratified by age, sex, ethnicity, TDI , hypertension, diabetes, body mass index (BMI), and smoking status, to investigate population-specific effects. Second, the association between OCT parameters and other neurodegenerative diseases was explored to assess the specificity of these markers. Logistic regression were performed to examine the relationship between baseline ocular diseases and the risk of developing amyotrophic lateral sclerosis, excluding individuals diagnosed during the follow-up period. Additionally, sensitivity analyses were performed by excluding individuals with follow-ups shorter than 4–6 years, removing individuals with ocular diseases**, and adjusting for smoking-related factors to minimize potential confounding effects and reduce the risk of reverse causality.**

Comment 9:

ABSTRACT

** Please confirm that your abstract complies with our requirements, including providing all the information relevant to this study type https://journals.plos.org/plosmedicine/s/submission-guidelines#loc-abstract*

** Please ensure that all numbers presented in the abstract are present and identical to numbers presented in the main manuscript text.*

** In the last sentence of the Abstract Methods and Findings section, please describe the main limitation(s) of the study's methodology.*

** l.30, "(year 2006-1010)" – please clarify that 2006-2010 only refers to baseline.*

** l.32, we suggest removing 'prospective'.*

** l.33: We think it would be helpful to state how many of the participants developed ALS (as done in the Results section of the main text).*

** Please include basic demographics, i.e. mean age, sex, ethnicity/race.*

** Please include the important dependent variables that are adjusted for in the analyses.*

** Please define the retinal markers investigated.*

Response: Thank you for your valuable comment. We have revised the abstract accordingly as follows (changes were marked in red):

Abstract

Background: Biomarkers are widely recognized as crucial breakthroughs in tackling amyotrophic lateral sclerosis (ALS). Among them, retina markers may hold promise due to the close retina-brain connection and non-invasive, portable detection methods. Thus, using optical coherence tomography (OCT), we investigated the link between baseline cell-level retinal features and future ALS risk.

Methods and Findings: Participants from the UK Biobank underwent OCT scans to assess retinal layers, macula, and optic disc parameters. Follow-up commenced two years after the baseline period (2006–2010), during which ALS cases were identified using International Classification of Diseases (ICD) codes from medical and assessment records. Cox proportional hazards models were applied to examine the relationship between retinal markers and incident ALS. Over a median follow-up of 14.11 years, 70 ALS cases occurred among 53,824 participants (incidence 10.58 per 100,000 person-years). Most participants were White (94.6%), 44.8% male, with a median age of 58 years. After adjusting for demographics and comorbidities affecting the retina, a standard deviation (SD) decrease of 15.19 μm in photoreceptor layer (PRL) thickness was associated with a 19% (95% confidence interval [7,29]; p=0.002) increased risk of ALS, while a SD increase of 26.11 μm in retinal pigment epithelium (RPE) thickness corresponded to a 20% (95% CI [7,34]; p=0.002) higher risk. Sensitivity analyses excluding follow-ups of less than 4 and 6 years yielded consistent results. Subgroup analyses showed these findings were more pronounced in smokers. The main limitation of this study is its single time-point observational design.

Conclusion: A thinner PRL and thicker RPE may precede the clinical diagnosis of ALS, offering potential clues for early diagnosis and insights into the disease's pathogenesis.

Comment 10: ***AUTHOR SUMMARY***

** The Author Summary requires revision: Each sub-heading should contain 2-3 single sentence, concise bullet points containing the most salient points from your study. Please revise accordingly. You may want to look at a recent publication to look at the formatting: https://doi.org/10.1371/journal.pmed.1004568*

** In the author summary, in the final bullet point of 'What Do These Findings Mean?', please include the main limitations of the study in non-technical language.*

Response: Thank you for the suggestion. The author summary has been revised accordingly as follows:

Why Was This Study Done?

- Amyotrophic lateral sclerosis (ALS) has limited diagnostic and treatment options, creating an urgent need for biomarkers to advance management.
- Retinal biomarkers may be emerging for ALS diagnosis due to retina-brain links and non-invasive detection.
- Current evidence on early retinal features in ALS is limited.

What Did the Researchers Do and Find?

- Cell-level retinal features were assessed using optical coherence tomography (OCT) data from 53,824 participants.
- Their association with future ALS risk was investigated over a median follow-up of 14.11 years.
- A thinner photoreceptor layer (PRL) and a thicker retinal pigment epithelium (RPE) were found to potentially precede the clinical diagnosis of ALS

What Do These Findings Mean?

- OCT-based assessment of PRL and RPE thickness may offer valuable clues for early ALS diagnosis and insights into its pathogenesis.
- The main limitation of this study is its single time-point observational design.

METHODS AND RESULTS

Comment 11:

** Please check that any use of statistical terms (such as trend or significant) are supported by the data, and if not please remove them. Please note that the term trend should be used only when the test for trend has been conducted.*

Response: Thank you for your suggestions. We have removed the "p for overall" from the manuscript and added details about the significance threshold and non-linearity testing in the Methods section (marked in red):

Method sections (Page 4, Line 151-163): All statistical analyses were performed using R software, version 4.2.1. The figures were created in Adobe Illustrator. A two-sided p-value less than 0.05 was considered significant; after Bonferroni correction, the significance threshold was adjusted to $p < 0.0056$.

Cox proportional hazards models were used to investigate the association between OCT parameters and incident ALS. Participants were censored at the date of ALS

diagnosis, death, or the end of follow-up, whichever occurred first. Non-linear relationships (p for non-linearity) were assessed using restricted cubic splines with three knots placed at the 10th, 50th, and 90th percentiles. Covariates, including age, sex, ethnicity, Townsend Deprivation Index (TDI), hypertension, diabetes, intraocular pressure, and spherical equivalent, were included (Supplementary methods in S2). Proportional hazard assumptions were evaluated using Schoenfeld residuals for all models (Supplementary Table 2 in S2). The multicollinearity analyses were assessed and showed Variance inflation factor (VIF) < 2.5 for independent covariates (Supplementary Table 3 in S2).

Comment 12: * *l.218: We think it would be useful to briefly mention that you did not observe any associations between RNFL, GCIPL, Macular thickness, Macular volume, Disc diameter and VCDR and ALS risk.*

Response: Thank you for the valuable suggestion. We have added the following statement to the manuscript:

Result sections (Page 5, Line 207-208): However, there were no associations between RNFL, GCIPL, macular thickness, macular volume, disc diameter, VCDR, and ALS risk.

Comment 13: * *Figure 1: Please revise the title, e.g. "Participant selection and study design" (or similar).*

Response: Thank you for the valuable suggestion. We have made the corresponding revision accordingly:

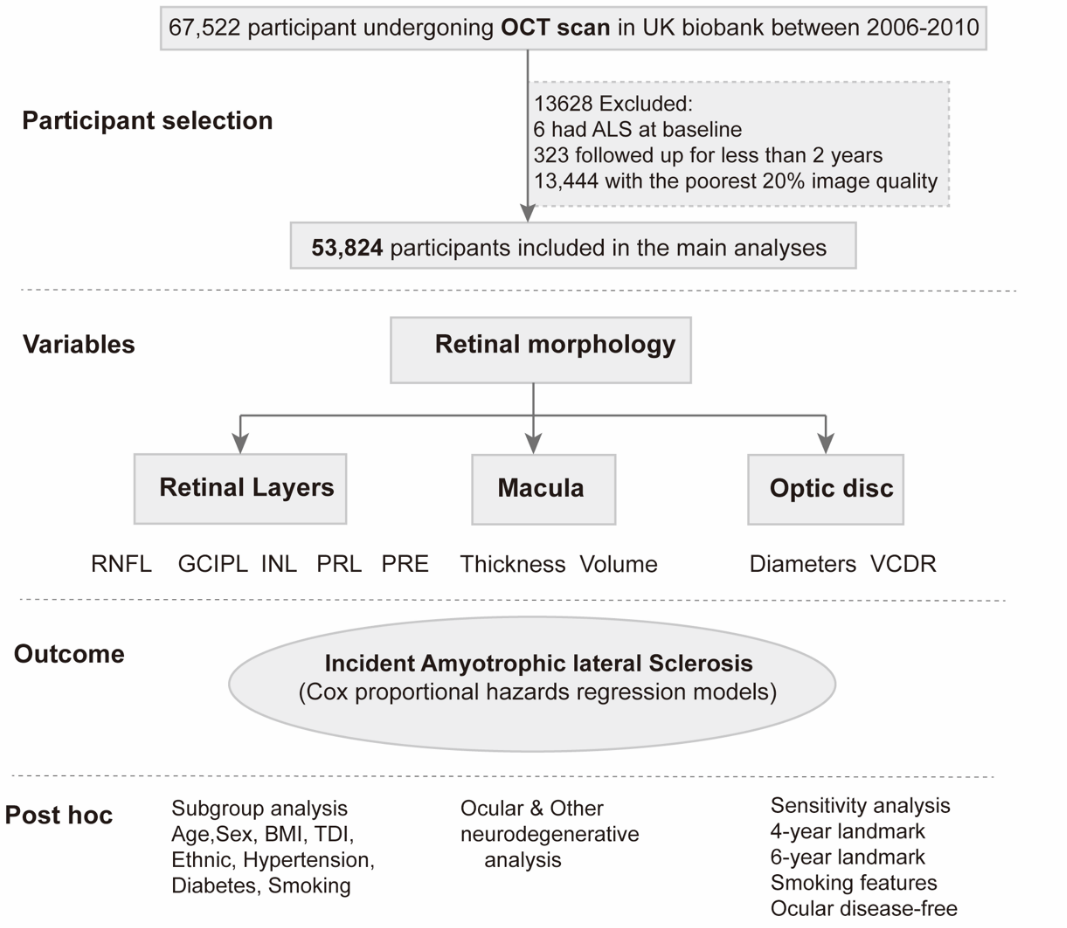

Figure 1. Participant selection and study design.
Abbreviation: OCT, Optical coherence tomography; TDI, Townsend Deprivation Index; BMI, Body mass index; RNFL, Retinal nerve fiber layer; GCIPLT, Ganglion cell-inner plexiform layer thickness; INL, Inner nuclear layer; PRL, Photoreceptor layer; RPE, Retinal pigment epithelium; VCDR, Vertical cup-to-disc ratio.

Comment 14: * *Figure 2: Please explain the meaning of the shaded area in the figure description. There's a typo in "Dis diameter". Also, the y-axis of the Disc Diameter graph seems lopsided and longer than the others. Please briefly mention the median time between marker assessment and ALS diagnosis.*

Response: Thank you for the valuable suggestion. We have made the corresponding revision accordingly.

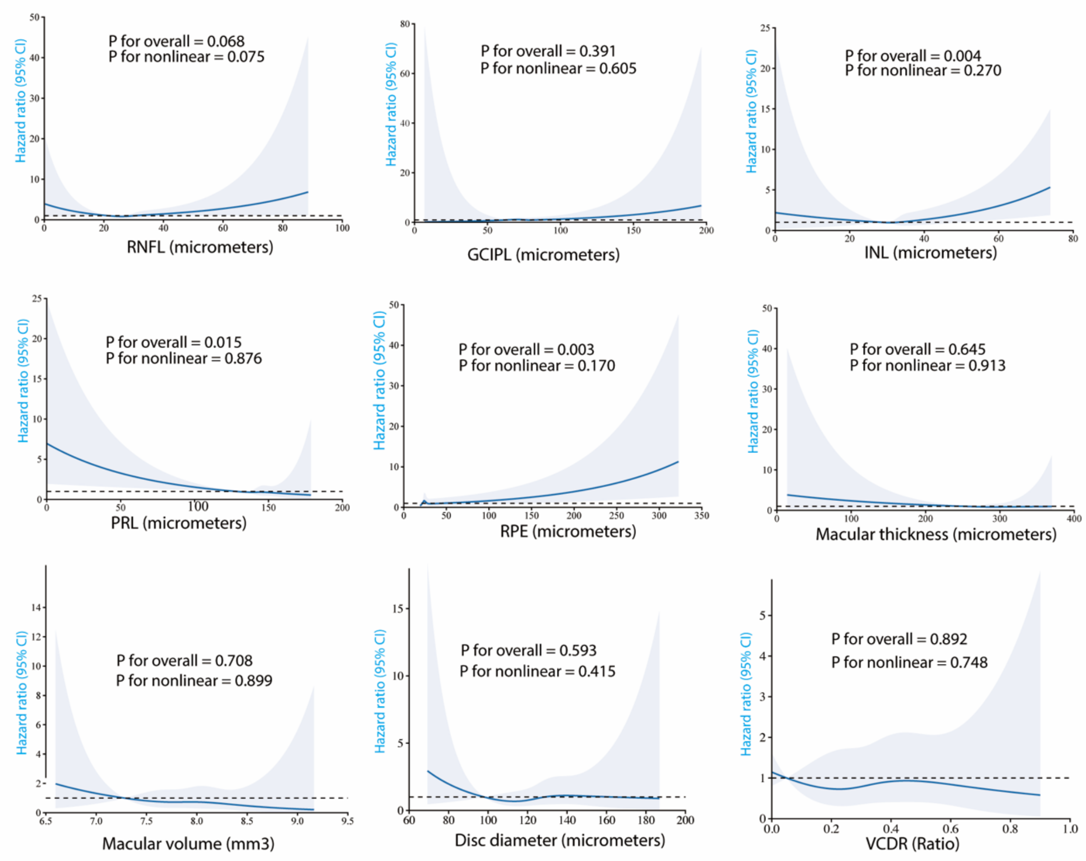

Figure 2. Non-linear relationship between retinal markers and incident ALS over a median follow up of 14.11 years.

Adjusted for age, sex, ethnicity, TDI, hypertension, diabetes, intraocular pressure, and spherical equivalent; Shaded area represents the 95% confidence interval.

Abbreviation: TDI, Townsend Deprivation Index; BMI, Body mass index; RNFL, Retinal nerve fiber layer; GCIPLT, Ganglion cell-inner plexiform layer thickness; INL, Inner nuclear layer; PRL, Photoreceptor layer; RPE, Retinal pigment epithelium; SD, Standard deviation; VCDR, Vertical cup-to-disc ratio.

Comment 15:* *Figure 3: We think it would be useful to briefly explain in the figure description what a TDI above or below 2.120 indicates. Please revise the figure title to be more specific. Please include the n numbers for each group.*

Response: Thank you for the valuable suggestion. We have made the corresponding revision accordingly.

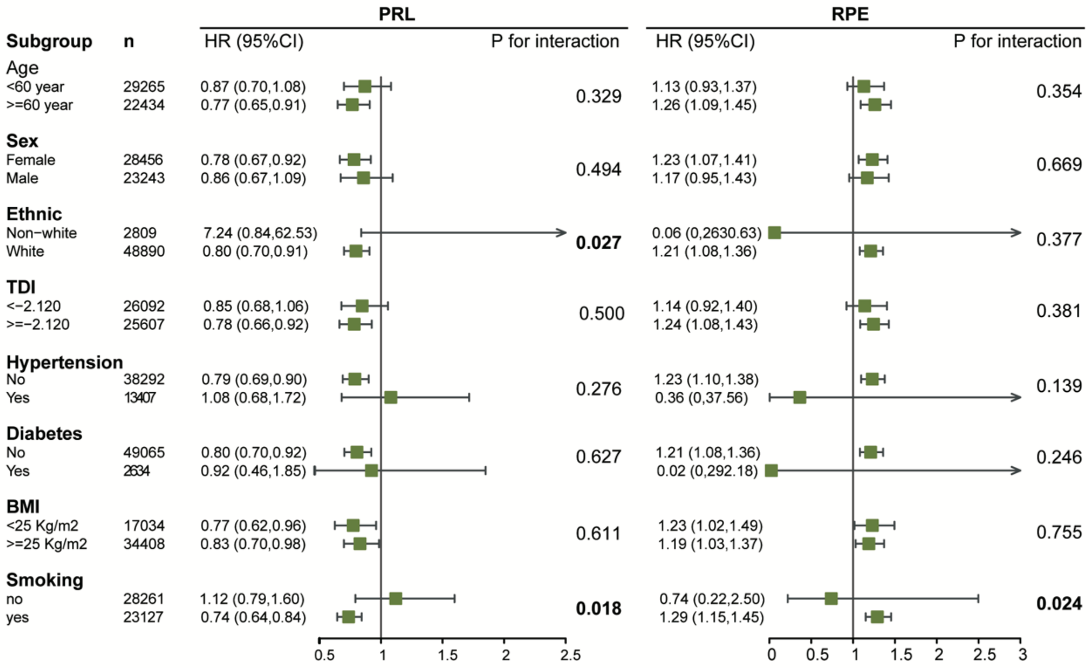

Figure 3. Subgroup analysis.
Adjusted for age, sex, ethnicity, TDI, hypertension, diabetes, intraocular pressure, and spherical equivalent;TDI was dichotomized at the median value (2.120), with higher values indicating greater socioeconomic deprivation.
Abbreviation: TDI, Townsend Deprivation Index; BMI, Body mass index; PRL, Photoreceptor layer; RPE, Retinal pigment epithelium;

Comment 16:* *Figure 4: Please note that figures should not normally be introduced in the Discussion section. We think it's fine to keep figure 4 in the discussion if you prefer.*

Response: Thank you for your comment. We agree with your point and appreciate the flexibility. We have retained Figure 4 in the Discussion section, as we believe it helps to clearly illustrate and support the interpretation of our findings.

Comment 17: * *Table 3: Please indicate the number of individuals for each group (i.e., each disease).*

Response: Thank you for the valuable suggestion. We have made the corresponding revision accordingly:

Table 3. Associations between OCT parameters and neurodegenerative diseases.

Index	ALS (case/n=70/53824)		AD (case/n=332/53511)		FTD (case=20/53514)	
	HR (95%CI)	p	HR (95%CI)	p	HR (95%CI)	p
Disc diameter (mm)	0.92 (0.70,1.20)	0.526	1.03 (0.90,1.18)	0.638	1.15 (0.68,1.93)	0.599
GCIPL (mm)	1.10 (0.90,1.35)	0.338	**0.78 (0.69,0.87)**	**0.000 ***	0.95 (0.56,1.64)	0.863
PRL (mm)	**0.81 (0.71,0.93)**	**0.002 ***	0.95 (0.86,1.05)	0.283	1.23 (0.92,1.66)	0.166
INL (mm)	**1.16 (1.04,1.29)**	**0.009**	0.95 (0.84,1.06)	0.352	0.94 (0.59,1.52)	0.804
RNFL (mm)	1.16 (0.88,1.53)	0.308	**0.85 (0.73,1.00)**	**0.045**	1.12 (0.63,1.98)	0.694
RPE (mm)	**1.20 (1.07,1.34)**	**0.002 ***	1.02 (0.91,1.14)	0.732	0.05 (0.00,6.44)	0.226
Macular thickness (mm)	0.89 (0.73,1.08)	0.239	**0.89 (0.81,0.97)**	**0.007**	1.18 (0.76,1.83)	0.470
Macular volume (mm^3)	0.82 (0.58,1.17)	0.279	**0.77 (0.64,0.91)**	**0.003 ***	0.86 (0.38,1.96)	0.719
VCDR (ratio)	0.94 (0.72,1.23)	0.660	**1.23 (1.08,1.40)**	**0.002 ***	0.82 (0.49,1.36)	0.436
**Index**	**PD** (case/n=328/53429)		**MSA** (case/n=13/53515)		**PSP** (case/n=22/53515)	
	HR (95%CI)	p	HR (95%CI)	p	HR (95%CI)	p
Disc diameter (mm)	0.97 (0.84,1.11)	0.622	0.51 (0.25,1.04)	0.063	0.93 (0.54,1.60)	0.785
GCIPL (mm)	**0.87 (0.77,0.99)**	**0.037**	0.66 (0.42,1.03)	0.067	1.09 (0.71,1.68)	0.702
PRL (mm)	0.94 (0.85,1.03)	0.179	1.08 (0.57,2.03)	0.819	**0.80 (0.64,0.99)**	**0.042**
INL (mm)	0.94 (0.84,1.05)	0.273	0.89 (0.53,1.49)	0.646	1.20 (0.99,1.45)	0.063
RNFL (mm)	0.98 (0.84,1.15)	0.808	0.62 (0.31,1.22)	0.166	1.18 (0.72,1.93)	0.504
RPE (mm)	0.99 (0.87,1.13)	0.897	0.61 (0.03,14.73)	0.763	1.21 (0.99,1.47)	0.060
Macular thickness (mm)	0.91 (0.83,1.01)	0.068	0.85 (0.57,1.27)	0.419	0.86 (0.63,1.19)	0.365
Macular volume (mm^3)	0.88 (0.74,1.05)	0.151	**0.36 (0.13,0.99)**	**0.047**	0.88 (0.39,1.98)	0.754

VCDR (ratio)	1.04 (0.91,1.19)	0.583	0.61 (0.31,1.18)	0.139	0.60 (0.35,1.03)	0.065

Adjusted for age, sex, ethnicity, TDI, hypertension, diabetes, intraocular pressure, and spherical equivalent; *significant after Bonferroni correction.

Abbreviation: ALS, Amyotrophic lateral sclerosis; AD, Alzheimer's disease; PD, Parkinson's disease; FTD, Frontotemporal dementia; MSA, Multiple system atrophy; PSP, Progressive supranuclear palsy:

TDI, Townsend Deprivation Index; BMI, Body mass index; IOP, Intraocular pressure; RNFL, Retinal nerve fiber layer; GCIPLT, Ganglion cell-inner plexiform layer thickness; INL, Inner nuclear layer; PRL, Photoreceptor layer; RPE, Retinal pigment epithelium; SD, Standard deviation; VCDR, Vertical cup-to-disc ratio; mm, micrometers.

General Editorial Requests

1) We ask every co-author listed on the manuscript to fill in a contributing author statement. If any of the co-authors have not filled in the statement, we will remind them to do so when the paper is revised. If all statements are not completed in a timely fashion this could hold up the re-review process. Should there be a problem getting one of your co-authors to fill in a statement we will be in contact. YOU MUST NOT ADD OR REMOVE AUTHORS UNLESS YOU HAVE ALERTED THE EDITOR HANDLING THE MANUSCRIPT TO THE CHANGE AND THEY SPECIFICALLY HAVE AGREED TO IT.

Response: Thank you for the reminder. All listed authors have completed their contributing author statements, and there have been no additions or removals of authors.

2) Please ensure that the paper adheres to the PLOS Data Availability Policy (see http://journals.plos.org/plosmedicine/s/data-availability), which requires that all data underlying the study's findings be provided in a repository or as Supporting Information. For data residing with a third party, authors are required to provide instructions with contact information for obtaining the data. PLOS journals do not allow statements supported by "data not shown" or "unpublished results." For such statements, authors must provide supporting data or cite public sources that include it.

Response: Thank you for the suggestion. The Data Availability Statement has been revised accordingly as follows:

The data supporting the findings of this study are available through the UK Biobank project site and require successful registration and application due to ethical and legal restrictions protecting participant privacy and data security. Further details on access procedures can be found at https://www.ukbiobank.ac.uk. Our research data derived from the UK Biobank dataset are available from the UK Biobank upon reasonable request. For data access inquiries, please contact the UK Biobank directly via their website or email: access@ukbiobank.ac.uk.

3) Please review your reference list to ensure that it is complete and correct. If you have cited papers that have been retracted, please include the rationale for doing so in the manuscript text, or remove these references and replace them with relevant current references. Any changes to the reference list should be mentioned in the rebuttal letter that accompanies your revised manuscript.

Response: We have thoroughly reviewed the reference list and confirm that it is complete and accurate. No retracted papers have been cited

Journal Requirements:

1) Please amend your detailed Financial Disclosure statement. This is published with the article, therefore should be completed in full sentences and contain the exact wording you wish to be published.
i). State what role the funders took in the study. If the funders had no role in your study, please state: "The funders had no role in study design, data collection and analysis, decision to publish, or preparation of the manuscript."
ii). If any authors received a salary from any of your funders, please state which authors and which funders.

Response: Thank you for your valuable comments. The financial disclosure has been updated accordingly. The current version reads:

This research was supported by the National Natural Science Foundation of China (Grant No. 81901273 to Binbin Deng, http://www.nsfc.gov.cn) and the Natural Science Foundation of Zhejiang Province (Grant No. ZCLY24H0903 to Binbin Deng, http://www.zjkjt.gov.cn). Professor Binbin Deng provided resources and designed the study. The funders had no role in study design, data collection and analysis, decision to publish, or preparation of the manuscript.

2) We note that your " Response to Reviewers file and MANUSCRIPT " files are duplicated on your submission. Please remove any unnecessary or old files from your revisio, and make sure that only those relevant to the current version of the manuscript are included.

Response: Thank you for pointing this out. We have removed the duplicate and outdated files, and only the relevant documents for the current version of the manuscript are now included in the submission.
3) Please send a completed 'Competing Interests' statement, including any COIs declared by your co-authors. If you have no competing interests to declare, please state "The authors have declared that no competing interests exist". Otherwise please declare all competing interests beginning with the statement "I have read the journal's policy and the authors of this manuscript have the following competing interests:"
Response: The authors have declared that no competing interests exist.

4) Thank you for your DAS statement " The data supporting the findings of this study are available on the UK Biobank project site and are subject to successful registration and application processes. Further details are available at https://www. UK biobank. ac=/. Our research data are available upon reasonable request "

In your Data Availability Statement, please ensure you have provided all necessary access information in complete sentences. Access information should include the name of any data repositories used as

well as digital object identifiers (DOIs), accession numbers or codes, or other persistent identifiers for all relevant data.

Response: Thank you for the suggestion. The Data Availability Statement has been revised accordingly as follows:

The data supporting the findings of this study are available through the UK Biobank project site and require successful registration and application due to ethical and legal restrictions protecting participant privacy and data security. Further details on access procedures can be found at https://www.ukbiobank.ac.uk. Our research data derived from the UK Biobank dataset are available from the UK Biobank upon reasonable request. For data access inquiries, please contact the UK Biobank directly via their website or email: access@ukbiobank.ac.uk.
